# Extracorporeal Photopheresis as a Possible Therapeutic Approach for Adults with Severe and Critical COVID-19 Non-Responsive to Standard Treatment: A Pilot Investigational Study

**DOI:** 10.3390/jcm12155000

**Published:** 2023-07-29

**Authors:** Bálint Gergely Szabó, Péter Reményi, Szabolcs Tasnády, Dorina Korózs, László Gopcsa, Marienn Réti, Andrea Várkonyi, János Sinkó, Botond Lakatos, János Szlávik, Gabriella Bekő, Ilona Bobek, István Vályi-Nagy

**Affiliations:** 1 Departmental Group of Infectious Diseases, Department of Haematology and Internal Medicine, Semmelweis University, H-1097 Budapest, Hungary; 2 South Pest Central Hospital, National Institute of Haematology and Infectious Diseases, Albert Florian út 5–7., H-1097 Budapest, Hungary

**Keywords:** SARS-CoV-2, severe acute respiratory syndrome coronavirus 2, COVID-19, coronavirus disease 2019, ECP, *extracorporeal photopheresis*, *pneumonia*, *pneumonitis*

## Abstract

**Background:** The optimal approach for adult patients hospitalized with severe and critical coronavirus disease 2019 (COVID-19), non-responsive to antiviral and immunomodulatory drugs, is not well established. Our aim was to evaluate feasibility and safety of extracorporeal photopheresis (ECP) in this setting. **Methods:** A prospective, single-center investigational study was performed between 2021 and 2022 at a tertiary referral center for COVID-19. Patients diagnosed with COVID-19 were screened, and cases with severe or critical disease fulfilling pre-defined clinical and biochemical criteria of non-response for >5 days, despite remdesivir, dexamethasone and immunomodulation (tocilizumab, baricitinib, ruxolitinib), were consecutively enrolled. After patient inclusion, two ECP sessions on two consecutive days per week for 2 weeks were applied. Patients were followed-up per protocol from study inclusion, and clinical, virological and radiological outcomes were assessed at the end of treatment (EOT) +28 days. **Results:** A total of seven patients were enrolled. At inclusion, four out of seven (57.1%) were admitted to the ICU, all patients had ongoing cytokine storm. Additionally, 3/7 (42.9%) had radiological progression on chest CT. At EOT+28 days, 2/7 (28.6%) patients died due to non-ECP-related causes. Among the survivors, no additional requirement for intensive care unit admission or radiological progression was observed, and invasive mechanical ventilation could be weaned off in 1/5 (20.0%). All patients achieved whole-blood SARS-CoV-2 RNAemia clearance, while 3/7 (42.9%) no longer showed detectable respiratory SARS-CoV-2 RNA. According to immune biomarker profiling, ECP mainly facilitated a decrease in plasma IL-6 and IL-17A levels, as well as the physiological regeneration of peripheral blood immunocyte subpopulations, notably CD8+/CD45RO+ memory T-cells. No safety signals were identified. **Conclusions:** ECP appears to be a safe and feasible option for adults hospitalized with severe or critical COVID-19 who do not respond to pharmacological interventions. Further trial data are warranted to assess its optimal use. **Trial registration:** ClinicalTrials.gov NCT05882331 (retrospectively registered).

## 1. Introduction

In adult patients requiring hospitalization for coronavirus disease 2019 (COVID-19), an acute viral illness of zoonotic origin caused by severe acute respiratory syndrome coronavirus 2 (SARS-CoV-2), a systemic hyperinflammation may develop. This state is characterized by persistent fever, arterial hypoxaemia, and multi-organ dysfunction involving the respiratory and cardiovascular system, the kidneys and the immune system [1,2,3,4,5,6,7]. As of June 2023, more than 6 million people have lost their lives due to confirmed COVID-19 since the start of the pandemic. In the search for better treatment options, several blood purification strategies, such as hemoadsorption, hemoperfusion and artificial liver blood purification, have been attempted in patients with severe and critical COVID-19, with mixed clinical results [8,9,10].

Extracorporeal photopheresis (ECP) is a non-toxic anti-inflammatory /immunomodulatory treatment. During ECP, patient blood is harvested, and then white blood cells are ex vivo separated and treated with 8-methoxypsoralen, a photoactive drug activated by ultraviolet-A irradiation. Subsequently, the cells are retransfused to the patient. Since its first use, ECP has been considered as an effective and safe strategy in conditions with a dysregulated adaptive immune response, such as acute cellular rejection in solid organ transplantation, graft-versus-host-disease in allogeneic hematopoetic stem-cell transplantation, and some systemic autoimmune disorders. It has been postulated that hyperactivated peripheral T-cells, which are representative of these pathophysiological states, are forced to enter apoptosis during ECP and are consequentially destroyed by tissue macrophages upon reintroduction to the body. The clearance of apoptotic T-cells also leads to the abatement of systemic inflammation by homeostatic reprogramming of macrophages, normalization of regulatory T-cells counts and type-1/type-2 helper T-cell ratios, and an establishment of anti-inflammatory cytokine milieu in peripheral blood [11]. Based on the published data, COVID-19 with systemic hyperinflammation is also characterized by a pathological decrease of regulatory T-cells and type-2 helper T-cell inhibition, along with an over-activation of tissue macrophages [12,13,14]. Therefore, we hypothesized that ECP may be beneficial for adults hospitalized with severe and critical COVID-19 who do not respond to other pharmacological treatments. 

## 2. Methods

### 2.1. Study Design

A prospective, single-center investigational study was performed between 2021 and 2022 at the South Pest Central Hospital, National Institute of Haematology and Infectious Diseases (Budapest, Hungary), a tertiary referral center with >250 beds for COVID-19 care. The study was conducted in accordance with the Declaration of Helsinki and national ethical standards. The study protocol was approved by the Institutional Review Board of South Pest Central Hospital, National Institute of Haematology and Infectious Diseases (IKEB-14/2020) and the Scientific and Research Ethics Committee of the Hungarian National Medical Scientific Council (ETT-TUKEB–IV/3937–1/2020/EKU). All patients provided written informed consent for anonymized data processing before study inclusion.

### 2.2. Patient Enrollment

Hospitalized adult patients (≥18 years at diagnosis) with diagnosed COVID-19 of any illness duration before hospitalization were eligible and screened for inclusion during daily on-site investigator visits. Patients were consecutively enrolled based on the following inclusion criteria: (1) severe or critical COVID-19, (2) clinical and biochemical non-response for >5 consecutive days, despite remdesivir, dexamethasone and immunomodulatory therapies (tocilizumab, baricitinib or ruxolitinib), with or without COVID-19 reconvalescent plasmatherapy, in absence of other plausible causes. Exclusion criteria were: (1) pregnancy or breastfeeding, (2) allergy or contraindications to 8-methoxypsoralen, (3) pre-COVID-19 ECP, (4) written informed consent was not obtainable. Clinical non-response was defined when ≥2 of the following were met compared to baseline: (1) persistent fever (non-contact tympanal measurement of >38.0 °C) for ≥48 h despite antipyretics, (2) progression of COVID-19 severity according to World Health Organization criteria by ≥1 stratum after ≥48 h, (3) persistent or worsening of partial arterial oxygen tension (PaO_2_)/inspired oxygen fraction (FiO_2_) index by ≥10% after ≥48 h despite respiratory support, (4) radiological progression by infiltrate extension on chest-computed tomography (CT) by ≥10% after ≥48 h, (5) new requirement of invasive mechanical ventilation, as determined necessary by an intensive care unit (ICU) team. Biochemical non-response was defined when ≥2 of the following laboratory analytes showed persistent or increasing levels by ≥20% after ≥48 h compared to baseline: (1) serum lactate dehydrogenase (LDH), (2) serum C-reactive protein (CRP), (3) serum ferritin, (4) plasma interleukin-6 (IL-6), (5) plasma D-dimer levels.

### 2.3. Data Collection

An electronic case report form was dedicated to the study purposes, and investigators manually uploaded anonymized patient data. The collected data during the study included: (1) age, sex, comorbidities and vaccination status, (2), symptom duration before diagnosis, (3) therapies administered for COVID-19, (4) outcomes and characteristics of follow-up (see below). Baseline data were recorded on the day of COVID-19 diagnosis. Inclusion data were registered on the day of the first ECP session.

### 2.4. Diagnostic and Therapeutic Strategies, Follow-Up

Symptoms of COVID-19 were defined according to the criteria of the European Centre for Disease Prevention and Control [15]. Diagnosis was based on the positivity of nasopharyngeal or endotracheal specimens for SARS-CoV-2 ribonucleic acid (RNA) using real-time polymerase chain reaction (RT-PCR). COVID-19 severity was determined based on the criteria of the World Health Organization [1]. Acute respiratory distress syndrome (ARDS) and acute respiratory failure were defined according to the Berlin criteria [16]. COVID-19-associated cytokine storm was previously defined by our group based on an interpretation published by Fajgenbaum et al. [17,18]. COVID-19 vaccination status was categorized as a primary series (two doses) or booster (≥1 additional dose), after ≥14 days of last vaccination with any of the vaccines authorized in Hungary (Janssen, Moderna, Oxford-AstraZeneca, Pfizer-BioNTech, Sinopharm-BBIBP-CorV, Sputnik-V).

Therapies against COVID-19 were allocated based on the disease severity following national and international guidelines [19,20]. The standard of care consisted of oxygen/respiratory support, intravenous fluids, antipyretics, antitussives, and bronchodilators as determined necessary by attending physicians. Patients requiring oxygen support received remdesivir (1 × 200 mg loading dose, 1 × 100 mg maintenance intravenously, 5 days minimum) and dexamethasone (1 × 6 mg orally, 5 days minimum). Either tocilizumab (8 mg/kg intravenously, single dose), baricitinib (1 × 4 mg orally, 7 days minimum) or ruxolitinib (2 × 5 mg orally, 7 days minimum) were administered to patients with a COVID-19-associated cytokine storm. The choice of therapy was based on drug accessibility, available route of administration and patient-specific contraindications. Patients also received COVID-19 reconvalescent plasmatherapy when compatible products were available.

Details of patient follow-up are demonstrated in Supplementary Table S1. Briefly, in-hospital follow-up of patients was conducted according to a standardized protocol from study inclusion until ECP end of treatment (EOT) plus 28 days or patient death. The EOT was declared after the last ECP session. During the study period, patients who were planned for discharge from the hospital before EOT+28 days were scheduled for follow-up by study investigators at our outpatient clinic at regular time intervals of 3 days. Physical examination, routine laboratory studies, arterial blood gas analyses were performed daily. Immune biomarker profiling, virological markers and chest CT scans were performed at baseline, inclusion, and on days EOT+3, EOT+7, EOT+14 and EOT+28. Immune biomarker profiling included a multi-cytokine analysis to measure concentrations of 20 cytokines/chemokines, as well as serum electrophoresis with immunofixation, immunoglobulins, complement-3/complement-4 and autoantibody levels, and fluorescence-activated cell sorting (FACS) of different subpopulations of T and B lymphocytes and natural killer (NK) cells from whole blood. Virological markers involved testing respiratory and whole-blood samples for the presence of SARS-CoV-2 nucleic acid by RT-PCR. All tests were performed according to the descriptions provided by the manufacturers.

### 2.5. Protocol of Extracorporeal Photopheresis

ECP was initiated on the day of study inclusion using the Therakos^TM^ Cellex^TM^ system, following the instructions of the manufacturer. Each patient underwent two ECP cycles for 2 weeks, with each cycle consisting of two sessions on 2 consecutive days per week (a total of four sessions), conducted through a peripheral or central venous access device. Cycles 1 and 2 were separated by 5 consecutive days. One ECP session took approximately 3 h to complete, and was divided into 4 phases: (1) during priming, the system performed a series of calibrations to ensure proper operation, (2) during collection, 1500 mL of whole blood was processed to collect a concentrated buffy coat containing white blood cells, while other cells and plasma were reinfused, (3) during the photoactive phase, a prescribed dose of 8-methoxypsoralen was added to the buffy coat, which was then circulated through ultraviolet-A photoactivation, (4) during reinfusion phase, treated cells were automatically reinfused to the patient. Multi-cytokine analysis and FACS for early and late apoptosis markers were performed from each ECP mononuclear cell collection (MNC), a sample type representative of the buffy coat.

### 2.6. Outcomes and Statistical Analysis

The clinical outcomes assessed were all-cause death, requirement for invasive mechanical ventilation and admission to the ICU. Virological outcomes included respiratory and whole-blood SARS-CoV-2 RT-PCR positivity. Radiological outcomes were evaluated based on radiological progression/regression or fixed infiltration on chest CT scan, measured by the relative extension of COVID-19 infiltration compared to the proportion of normal lung tissue. All outcomes were assessed at EOT+28 days and compared to data at inclusion. Continuous variables are expressed as median ± interquartile ranges and minimum–maximum values; categorical values are reported in absolute numbers and relative percentages. Statistical comparisons were performed using Fisher’s exact-test or the Mann–Whitney U-test. A two-tailed *p* value of <0.05 determined statistical significance. For reporting, we adhered to the “Better reporting of interventions: template for intervention description and replication” (TIDieR) checklist (http://www.tidierguide.org, accessed on 1 March 2023).

## 3. Results

### 3.1. Demographic Characteristics of Patients

The study flowchart is presented in Supplementary Figure S1. During the study period, 1035 eligible patients were screened. Among them, 296 patients had severe or critical COVID-19, and 7 met study inclusion criteria. Demographic data and therapeutic strategies are reported in Table 1.

Median age was 54 ± 14 (32–72) years, with a male dominance in the cohort (5/7, 71.4%). Median duration of symptom before diagnosis was 2 ± 4 (1–7) days. Prevalent comorbidities included essential hypertension (5/7, 71.4%), chronic cardiac and pulmonary diseases (3/7, 42.9% each). Four patients received the primary series of COVID-19 vaccinations, and two also received boosters. 

### 3.2. Clinical Characteristics of Patients

Clinical characteristics are reported in Table 2.

At baseline, 4/7 (57.1%) patients had severe COVID-19, with a median infiltrate extension of 50 ± 60% (20–80) on chest CT. Cytokine storm was documented in six cases (85.7%), 3/7 (42.9%) patients had ARDS. Two patients were admitted to the ICU, requiring invasive mechanical ventilation (28.6%). At inclusion, all patients had ongoing cytokine storm, and an additional patient progressed to critical severity (5/7, 71.4% in total). Three (42.9%) more patients showed radiological progression on chest CT. An additional two patients had to be admitted to the ICU, without any further ARDS development (4/7, 57.1% in total).

### 3.3. Laboratory Characteristics of Patients

Laboratory characteristics are reported in Table 3. 

From baseline to inclusion, an increase in the absolute neutrophil count (ANC) with a median of 1.5 ± 5.9 × 10^9^/L and an absolute lymphocyte count (ALC) with a median of 0.7 ± 0.4 × 10^9^/L, along with serum ferritin (381 ± 230 μg/L) and LDH (153 ± 96 UI/L) was documented, while serum CRP decreased with a median of 75 ± 14 mg/L (*p* > 0.05 for all). At follow-up, ANC and ALC, serum ferritin, CRP and plasma D-dimer values showed a statistical trend towards their reference ranges (*p* > 0.05 for all), while serum LDH declined significantly (1096 ± 379 vs. 522 ± 214 UI/L, *p* = 0.02). 

### 3.4. Immune Biomarker Profiling of Patients

Results of immune biomarker profiling are reported in Table 4.

At inclusion, multi-cytokine analysis revealed an increasing trend of serum RANTES, plasma interleukin- (IL-) 4 and IL-6 levels compared to baseline (*p* > 0.05 for all differences). During follow-up, both plasma IL-6 (203.5 ± 1688.0 vs. 6.5 ± 24.0 pg/mL, *p* = 0.04) and interleukin-17A (4.1 ± 3.8 vs. 1.3 ± 0.1 pg/mL, *p* < 0.01) levels decreased significantly, while the concentrations of serum interferon- (IFN-) γ and IP-10, and plasma IL-5, IL-8 and IL-10 showed trends of increase. Complement consumption or paraprotein/autoantibody generation was not documented among patients. Peripheral whole-blood FACS parameters are reported in Table 5. 

From baseline to inclusion, a statistically non-significant decrease among CD3+, CD3+/CD8+ and CD3+/CD4+ T, CD3-/CD16+/CD56+ NK and CD19+ B-cell subpopulations, with an increase in double-negative T-cell ratios, CD3+/TCRγδ+, CD8+/CD45RO+ and CD4+/CD45RO+ memory T-cell counts was documented (*p* > 0.05 for all differences). During follow-up, double-negative T-cell ratios, CD3+/TCRγδ+ and CD8+/CD45RA+ naive T-cell numbers decreased. Other lymphoid subpopulations showed a trend for physiological regeneratior, with a statistically significant increase in CD8+/CD45RO+ memory T-cell counts (76.2 ± 94.1 vs. 144.4 ± 127.0 cells/μL, *p* = 0.03).

### 3.5. Outcome Characteristics of Patients

Outcomes and therapeutic strategies against COVID-19 are reported in Table 1. Two (28.6%) patients died due to septic shock unrelated to ECP (*Staphylococcus aureus* and *Klebsiella pneumoniae* bloodstream-infections) at EOT+22 and EOT+24 days, respectively. Invasive mechanical ventilation could be weaned in one, and had to be continued in two patients. The rate of ICU admission requirement (57.1%) among patients did not increase at EOT+28 days compared to inclusion. During follow-up, all patients reached peripheral blood clearance of SARS-CoV-2 RNAemia, while respiratory SARS-CoV-2 PCR positivity remained detectable in four patients (57.1%). Radiological progression was only found in patients with a fatal outcome. The median length of stay was 67 ± 75 (22–155) days. Both tocilizumab and baricitinib were administered to four patients each (57.1% each). Reconvalescent plasmatherapy could be administered to six patients (85.7%).

### 3.6. ECP MNC Apoptosis Assessment 

FACS with early/late apoptosis markers and multi-cytokine analysis from ECP MNC are reported in Supplementary Table S2 and Supplementary Material S1. During ECP, 35–51% of lymphocytes, 38–47% of T-cells, and 95–97% of monocytes entered the early apoptotic stage. Additionally, from the first to last MNC collections, there was a statistically nonsignificant trend towards rising the rates of cells entering early apoptosis, a phenomenon more pronounced among lymphocytes and T-cells. Necrotic cells were not detected, while rates of lymphocytes, T-cells and monocytes entering late apoptotic stage remained low. Cytokines were detected in both increasing (IL-1α, IL-10 and IL-15) and decreasing (IFN-α, IL-5, IL-6, IL-8, IL-13, IP-10, MIP-1α, TNF-β) concentrations, while other cytokines did not show relevant fluctuations (IFN-γ, IL-1β, IL-2, IL-4, IL-12, IL-17A, MCP-1, RANTES, TNF-α) in serial samples.

## 4. Discussion

### 4.1. Main Study Findings

To the best of our knowledge, this is the first study assessing feasibility and safety of extracorporeal photopheresis among adults hospitalized for severe and critical COVID-19 who showed non-responsiveness to pharmacological treatment modalities. Our hypothesis was that ECP might attenuate the systemic hyperinflammation associated with COVID-19 in this patient population.

Seven mostly middle-aged male patients with a significant comorbidity burden requiring oxygen supportation and showing prolonged clinical and biochemical non-responsiveness, despite the administration of remdesivir, dexamethasone and immunomodulation (tocilizumab, baricitinib, ruxolitinib), were enrolled in our pilot investigational study. At inclusion, the majority of patients had to be admitted to the ICU in critical condition, with an ongoing COVID-19-associated cytokine storm and progression of infiltrates on chest CT scans. At EOT+28 days, two patients died due to non-ECP-related sepsis. Among those who survived, invasive mechanical ventilation could be discontinued in one patient; additional patients did not require ICU admission, and further radiological progression was not observed on chest CT scans. All the included patients cleared SARS-CoV-2 RNAemia, while a minority also lost respiratory SARS-CoV-2 PCR detectability.

During ECP cycles, it was proven that 35–50% of all lymphocytes and T-cells, as well as almost all monocytes entered the early apoptotic phase. Specific cytokine fluctuations pointed towards an evolving anti-inflammatory milieu, further supporting our hypothesis. After ECP, blood absolute lymphocyte counts, serum ferritin, CRP, LDH and plasma D-dimer levels showed a trend towards their physiological reference ranges. ECP also decreased plasma concentrations of IL-6 and IL-17A, while the regeneration of peripheral blood immunocyte subpopulations, including CD3+/CD8+ and CD3+/CD4+ T-cells, CD3-/CD16+/CD56+ NK-cells and CD19+ B-cells, was documented with a significant increment in CD8+/CD45RO+ memory T-cell counts. No biochemical or clinical safety signals for ECP were identified during the study period.

### 4.2. Previous Literature Findings

Since the start of the pandemic, the literature evidence has been suggesting that a dysregulated cellular and humoral host immune response could aggravate the severity of COVID-19, leading to critical organ dysfunction with poor disease prognosis [12].

In one of the earliest studies, 435 patients with confirmed SARS-CoV-2 infection were followed for 6 weeks. Counts of all measured lymphocyte subpopulations, including CD3+, CD4+, CD8+, CD19+ and CD16/56+ cells, remained below their respective reference ranges for ≥5 weeks. In addition, severe disease and fatal outcomes were associated with pronounced lymphopenia in peripheral blood compared to non-severe cases and survivors [13]. These findings were mirrored in other studies, mostly investigating patients with severe COVID-19, and suggested that peripheral neutrophil-to-lymphocyte ratios, and especially neutrophil-to-CD8+ T-cell ratios, as well as absolute CD8+ T-cell counts correlated well with poor clinical outcomes, including mortality [21,22]. A later study proved that the acute immunodeficiency character of COVID-19 stems from the immunoparesis of the CD4+ T-cell pool, and can also be demonstrated by peripheral T-cell exhaustion [23]. Other authors have also highlighted CD4+ T-cell peripheral depletion and altered functionality as negative prognostic markers for severity and survival in COVID-19 [24,25]. In the context of COVID-19 pulmonary infiltrations, histopathological evidence gathered from 60 autopsies demonstrated that CD8+ T-cells were abundant, whereas the CD4+ T cell presence was low in the pulmonary interstitium, in contrast to the cases of control patients without infection [26]. These findings were later mirrored by an *in vivo* clinical study which proved a correlation between CD4+ T-cell lymphopenia and pulmonary inflammation on chest CT [27,28]. We note that other cellular subpopulations also play a role in disease progression. For example, it has been shown that phenotypically distinct CD3+/TCRγδ+, double-negative, and double-positive T-cells proliferate during the acute stage of SARS-CoV-2 infection, parallel to prominent expansions of CD14+ classical and CD16+ non-classical monocyte subpopulations. While CD4+/CD45RA+ and CD8+/CD45RA+ naive T-cells, as well as CD4+/CD45RO- central memory T-cells decreased in number, the number of CD8+/CD45RO+ and CD4+/CD45RO+ effector memory T-cells increased, compared to healthy controls and reconvalescent patients alike, strengthening the idea that abnormal peripheral T-cell activation and differentiation are key steps in SARS-CoV-2 pathophysiology [29].

On the other hand, dysregulated cytokine responses, most notably fluctuations of serum IL-6 during COVID-19, have also been established in the literature [17]. Among critically ill patients with COVID-19, serum IL-6 and IL-10 levels are usually higher, while serum GM-CSF, TNF- α, IFN-γ, IL-2 and IL-8 serve as ancillary cytokines with clinical prognostic value in contrast to patients without critical disease [14,30,31]. Since tocilizumab is recommended for patients with COVID-19 of critical severity, its effect on cytokine levels should also be mentioned. For example, in a study carried out by Azmy et al., post-tocilizumab serum IL-6 levels spiked and then showed a steady decrease, while the levels of the anti-inflammatory IL-10 were not affected [31]. Our group previously documented similar trends of serum IL-6 among critically ill patients receiving immunomodulatory therapies [18,32]. Other studies have demonstrated that persistently elevated serum IL-6 levels during COVID-19 are independently associated with in-hospital mortality, and rises of serum IL-6, IL-8 and TNF-α are probably age-dependent, translating to poorer outcomes among the elderly [22,24,33]. Pro-inflammatory cytokine responses in blood were detectable for ≥3 weeks after SARS-CoV-2 infection [34]. In addition, serum levels of both IL-6 and IL-10 correlated positively with pulmonary lesion volumes on chest CT scans caused by COVID-19, while others argued that the TNF-α/IL-10 ratio could readily estimate progression to acute respiratory failure during COVID-19 [27,35]. Lastly, hyperinflammation during COVID-19 is probably accompanied by a simultaneous anti-inflammatory immune response driven by serum IL-10, which therefore also bears a deterministic effect on disease severity [36]. 

During cytokine storm, the complex interplay between immunocytes and secreted cytokines is obvious. For example, serum IL-6 and IL-8 levels correlate well with CD4+ and CD8+ T-cell counts and ongoing pulmonary inflammation [28]. Furthermore, blood levels of T-cell-associated cytokines, mainly IFN-γ, TNF-α, IL-2, IL-6 and IL-17, increase during severe COVID-19, as opposed to measurements in controls [23]. Lung samples from COVID-19 autopsies revealed that the inflamed pulmonary endothelium expressed IL-1β, IL-6, IL-15 and TNF-α, in contrast to non-infected tissue samples, recruiting inflammocytes along the alveolar epithelium [26]. Overall, while much has been discovered about COVID-19-associated inflammation, a therapeutic strategy for COVID-19 non-responsive to antiviral and immunomodulatory drugs has not yet been established in the literature. Therefore, given the high level of cellular and humoral immune dysregulation accompanying severe and critical COVID-19, non-toxic modalities aiming to reduce inflammation-related multi-organ damage, such as extracorporeal photopheresis, are probably crucial for clinical practice.

### 4.3. Limitations

A comparator arm was not feasible for this study. Since this was a pilot study, the number of enrolled patients is low. Despite limitations, we feel that results might serve as a basis for future research and possibly introduce a novel therapeutic alternative for a vulnerable population with limited treatment options and an overall negative prognosis.

## 5. Conclusions

Extracorporeal photopheresis might be considered a feasible, safe novel treatment for adults hospitalized with severe and critical COVID-19 who do not respond to other pharmacological therapies. Further research is warranted to confirm the findings of this pilot study.

## Figures and Tables

**Table 1:** Demographic data, therapeutic strategies and outcomes of patients enrolled in the study.

Patient Identification	Pt. 1.	Pt. 2.	Pt. 3.	Pt. 4.	Pt. 5.	Pt. 6.	Pt. 7.	Total
**Age (years)**	55	65	32	54	51	41	72	54 ± 14 (32–72)
**Gender (n, %)**	M	M	M	M	M	F	F	M: 5/7 (71.4) F: 2/7 (28.6)
**Comorbidities (n, %)**	AC	HT OB	CCD CHD CRD CVD HT	CPD HEM TOB	CPD HT OB	AID CHD CPD CVD HT TOB	AID CHD CRD CVD HEM HT	AC: 1/7 (14.3) AID: 2/7 (28.6) CCD: 1/7 (14.3) CHD: 3/7 (42.9) CPD: 3/7 (42.9) CRD: 2/7 (28.6) CVD: 3/7 (42.9) HEM: 2/7 (28.6) HT: 5/7 (71.4) OB: 2/7 (28.6) TOB: 2/7 (28.6)
**COVID-19 vaccination status:**								
- Primary series (n, %)	N	2 × AZ	2 × Sp	2 × Pf	2 × Pf	2 × Pf	2 × Pf	Y: 6/7 (85.7)
- Booster vaccination (n, %)	n.a.	1 × Pf	N	N	N	N	1 × Pf	Y: 2/6 (33.3)
- Time since last vaccine (months)	n.a.	1	8	7	6	6	2	6 ± 5 (1–8)
**Symptom duration before** **COVID-19 diagnosis (days)**	7	6	1	1	2	2	4	2 ± 4 (1–7)
**Therapies against COVID-19 (n, %):**								
- Remdesivir	Y	Y	Y	Y	Y	Y	Y	Y: 7/7 (100)
- Dexamethasone	Y	Y	Y	Y	Y	Y	Y	Y: 7/7 (100)
- Tocilizumab	Y	Y	Y	N	N	Y	N	Y: 4/7 (57.1)
- Baricitinib	Y	Y	N	N	Y	N	Y	Y: 4/7 (57.1)
- Ruxolitinib	N	N	N	Y	N	N	N	Y: 1/7 (14.3)
- Reconvalescent plasmatherapy	Y	N	Y	Y	Y	Y	Y	Y: 6/7 (85.7)
**Clinical outcomes (n, %):**								
- All-cause death	N	N	N	N	N	Y	Y	Y: 2/7 (28.6)
- Invasive mechanical ventilation	Y	N	Y	N	N	N *	N *	Y: 2/7 (28.6)
- ICU admission	Y	N	Y	N	Y	N *	Y *	Y: 4/7 (57.1)
**Virological outcomes (n, %):**								
- Respiratory SARS-CoV-2 RT-PCR positivity	N	Y	N	N	Y	Y *	Y *	Y: 4/7 (57.1)
- Blood SARS-CoV-2 RT-PCR positivity	N	N	N	N	N	N *	N *	Y: 0 (0)
**Radiological outcomes (n, %)**								
- Radiological progression on chest CT scan	N	N	N	N	N	Y *	Y *	Y: 2/7 (28.6)
- Radiological regression on chest CT scan	N	Y	N	Y	Y	N	N	Y: 3/7 (42.9)
- Fixed infiltration on chest CT scan	Y	N	Y	N	N	N	N	Y: 2/7 (28.6)
Hospital ward length of stay (days) **	86	45	0	30	51	24	0	45 ± 42 (24–86)
**Intensive care unit** **length of stay (days)**	69	0	91	0	48	0	22	59 ± 45 (22–91)
**Time to death from hospital admission (days)**	n.a.	n.a.	n.a.	n.a.	n.a.	24	22	23 ± n.a. (22–24)

* Outcome assessed on day of patient death by last available data. ** Excluding length of stay at the intensive care unit. AC: chronic alcohol consumption, AID: systemic autoimmune disease, ARDS: acute respiratory distress syndrome, AZ: Oxford–AstraZeneca COVID-19 vaccine, CCD: chronic cerebral disease, CHD: chronic heart disease, CPD: chronic pulmonary disease, CRD: chronic renal disease, CT: computed tomography, CVD: chronic vascular disease, F: female, HEM: active hematological malignancy, HT: essential hypertension, ICU: intensive care unit, M: male, N: no, n.a.: not applicable, OB: obesity, Pf: Pfizer–BioNTech COVID-19 vaccine, RNA: ribonucleic acid, SARS-CoV-2: severe acute respiratory syndrome *coronavirus 2*, Sp: Sputnik-V COVID-19 vaccine, TOB: chronic tobacco use, Y: yes.

**Table 2:** Clinical characteristics at baseline and inclusion of patients enrolled in the study.

Patient Identification	Pt. 1.		Pt. 2.		Pt. 3.		Pt. 4.		Pt. 5.		Pt. 6.		Pt. 7.		Total		*p* Value **
Time of Assessment	Baseline	Inclusion	Baseline	Inclusion	Baseline	Inclusion	Baseline	Inclusion	Baseline	Inclusion	Baseline	Inclusion	Baseline	Inclusion	Baseline	Inclusion	
**COVID-19 severity**	C	C	C	C	C	C	S	S	C	C	S	C	S	S	C: 4/7 (57.1) S: 3/7 (42.9)	C: 5/7 (71.4) S: 2/7 (28.6)	1.0
Infiltrate extension on chest CT (%) *	80	80	50	50	80	80	20	30	80	80	20	80	20	50	50 ± 60 (20–80)	80 ± 30 (30–80)	0.37
**Partial arterial oxygen tension** per fraction of inspired oxygen (mmHg/%) *	95	85	175	80	115	90	300	200	55	90	205	85	300	200	175 ± 205 (55–300)	90 ± 115 (80–200)	0.16
**ARDS**	Y	Y	N	N	Y	Y	N	N	Y	Y	N	N	N	N	Y: 3/7 (42.9)	Y: 3/7 (42.9)	1.0
**Cytokine storm**	Y	Y	Y	Y	Y	Y	Y	Y	Y	Y	N	Y	Y	Y	Y: 6/7 (85.7)	Y: 7/7 (100%)	1.0
**Respiratory SARS-CoV-2** **RT-PCR positivity**	Y	Y	Y	Y	Y	Y	Y	Y	Y	Y	Y	Y	Y	Y	Y: 7/7 (100%)	Y: 7/7 (100%)	1.0
**Blood SARS-CoV-2** **RT-PCR positivity**	Y	Y	N	Y	Y	Y	N	Y	Y	Y	N	N	N	Y	Y: 3/7 (42.9)	Y: 6/7 (85.7)	0.31
**ICU admission**	N	Y	N	N	Y	Y	N	N	Y	Y	N	N	N	Y	Y: 2/7 (28.6)	Y: 4/7 (57.1)	0.59
**Invasive mechanical ventilation**	N	Y	N	N	Y	Y	N	N	Y	Y	N	N	N	N	Y: 2/7 (28.6)	Y: 3/7 (42.9)	1.0

* Parameters are reported in median ± interquartile ranges and minimum–maximum values. ** *p* values are calculated between cumulated data, measured at baseline and inclusion. ARDS: acute respiratory distress syndrome, C: critical COVID-19, CT: computed tomography, E: extracorporeal photopheresis, ICU: intensive care unit, N: no, RNA: ribonucleic acid, S: severe COVID-19, SARS-CoV-2: severe acute respiratory syndrome *coronavirus 2*, Y: yes.

**Table 3:** Routine laboratory parameters of patients enrolled in the study at baseline, inclusion and post-ECP follow-up.

Parameter	Baseline	Inclusion	*Post*-ECP Follow-Up				*p* Value *
			+3 Days	+7 Days	+14 Days	+28 Days	
**Blood absolute white blood** **cell count (×10^9^/L)**	8.1 ± 3.2 (2.1–10.5)	8.9 ± 7.4 (1.9–21.3)	14.3 ± 7.4 (3.7–23.5)	8.1 ± 2.4 (4.5–21.4)	6.4 ± 6.4 (2.3–19.7)	8.1 ± 2.5 (3.7–9.2)	0.97
**Blood absolute neutrophil** **granulocyte count (×10^9^/L)**	4.7 ± 2.0 (1.8–9.2)	6.2 ± 7.9 (1.4–16.9)	8.2 ± 6.9 (2.5–20.1)	6.1 ± 4.6 (3.0–17.7)	3.8 ± 5.1 (1.1–15.5)	4.4 ± 1.9 (2.1–6.1)	0.46
**Blood absolute lymphocyte** **count (×10^9^/L)**	0.8 ± 1.7 (0.1–4.1)	1.5 ± 1.3 (0.2–3.3)	1.3 ± 1.1 (0.4–5.4)	1.4 ± 1.7 (0.5–3.6)	1.4 ± 1.2 (0.3–2.6)	1.8 ± 1.6 (0.4–6.0)	0.52
**Blood absolute monocyte** **count (×10^9^/L)**	0.3 ± 0.2 (0.1–0.8)	0.3 ± 0.3 (0.2–1.5)	0.6 ± 0.5 (0.1–1.4)	0.4 ± 0.3 (0.1–1.7)	0.5 ± 0.7 (0.3–2.0)	0.7 ± 0.6 (0.3–0.9)	0.7
**Blood hemoglobin (g/L)**	113 ± 18 (96–141)	136 ± 40 (86–140)	111 ± 31 (76–142)	98 ± 19 (82–131)	96 ± 13 (84–133)	85 ± 12 (75–139)	0.07
**Blood platelet count (×10^9^/L)**	177 ± 113 (49–368)	302 ± 208 (43–853)	230 ± 233 (26–432)	167 ± 220 (30–498)	111 ± 126 (43–705)	156 ± 368 (35–783)	0.87
**Serum lactate dehydrogenase (IU/L)**	943 ± 283 (400–1168)	1096 ± 379 (530–1802)	807 ± 200 (586–2020)	693 ± 166 (505–847)	473 ± 88 (389–626)	522 ± 214 (274–626)	**0.02**
**Serum glutamic pyruvate** **transaminase (IU/L)**	34 ± 16 (11–48)	31 ± 39 (18–76)	75 ± 41 (19–395)	85 ± 71 (16–253)	119 ± 192 (19–338)	38 ± 56 (16–117)	0.62
**Serum creatinine (μmol/L)**	117 ± 134 (64–364)	127 ± 97 (61–315)	93 ± 38 (46–371)	113 ± 178 (33–317)	98 ± 80 (35–281)	68 ± 57 (29–120)	0.1
**Serum ferritin (μg/L)**	1107 ± 1340 (35–2478)	1488 ± 1110 (249–6241)	1185 ± 2122 (284–4034)	1709 ± 1895 (470–3052)	822 ± 1247 (444–2439)	715 ± 505 (324–3753)	0.74
**Serum C-reactive protein (mg/L)**	114 ± 142 (33–279)	39 ± 128 (1–245)	30 ± 50 (4–94)	44 ± 59 (2–192)	43 ± 160 (0–338)	35 ± 3 (0–46)	0.28
**Serum procalcitonin (ng/mL)**	0.1 ± 0.1 (0–0.1)	0.1 ± 0.1 (0–1.2)	0 ± 0.1 (0–1.3)	0.1 ± 0.1 (0–2.1)	0.2 ± 0.6 (0–0.7)	0 ± 0 (0–2.4)	0.92
**Plasma D-dimer (ng/mL)**	1184 ± 568 (642–2128)	1254 ± 1601 (659–3581)	1145 ± 327 (799–1473)	1411 ± 672 (496–2384)	1592 ± 1108 (261–13920)	763 ± 250 (597–965)	0.21

* *p* values are calculated between data measured at inclusion and +28 days *post*-ECP.

**Table 4:** Immune biomarker profiling of patients enrolled in the study at baseline, inclusion and post-ECP follow-up.

Parameter	Baseline	Inclusion	*Post*-ECP Follow-Up				*p* Value *
			+3 Days	+7 Days	+14 Days	+28 Days	
Serum interferon-α (pg/mL)	33.6 ± 25.8 (8.0–60.4)	14.8 ± 11.8 (1.5–34.7)	9.0 ± 3.3 (1.5–47.3)	9.0 ± 10.5 (8.0–60.4)	8.0 ± 0.3 (8.0–9.0)	8.0 ± 0.1 (8.0–14.8)	0.14
Serum interferon-γ (pg/mL)	7.7 ± 25.5 (1.3–48.6)	1.3 ± 34.5 (1.3–37.7)	10.0 ± 16.0 (1.3–44.7)	1.3 ± 31.6 (1.3–148.3)	7.4 ± 20.0 (1.3–44.9)	6.0 ± 4.9 (1.3–22.1)	0.33
Plasma interleukin-1α (pg/mL)	40.7 ± 23.6 (4.8–67.4)	23.3 ± 16.3 (4.8–91.9)	55.7 ± 45.0 (4.8–68.2)	34.9 ± 48.0 (4.8–68.2)	20.4 ± 156.1 (4.8–535.2)	8.2 ± 55.9 (4.8–60.7)	0.18
Plasma interleukin-1β (pg/mL)	7.2 ± 9.0 (1.6–11.9)	1.6 ± 3.8 (1.6–9.2)	4,2 ± 5,8 (1.6–10.6)	1.6 ± 4.6 (1.6–40.1)	1.9 ± 1.6 (1.6–6.2)	1.6 ± 0.1 (1.6–1.7)	0.12
Plasma interleukin-2 (pg/mL)	1.0 ± 1.1 (0.6–10.6)	0.6 ± 0.7 (0.6–2.2)	0.6 ± 0.8 (0.6–6.9)	0.6 ± 0.1 (0.6–16.8)	0.8 ± 1.1 (0.6–4.5)	0.6 ± 0.3 (0.6–1.7)	1.0
Plasma interleukin-4 (pg/mL)	2.2 ± 6.3 (0.9–15.2)	3.2 ± 2.8 (0.6–10.1)	1.7 ± 4.6 (0.6–6.2)	1.0 ± 10.5 (0.6–45.2)	2.6 ± 5.9 (0.6–12.6)	1.3 ± 2.1 (0.6–8.4)	0.44
Plasma interleukin-5 (pg/mL)	4.7 ± 21.7 (0.6–44.9)	2.7 ± 1.4 (0.6–3.0)	1.2 ± 0.7 (1.0–5.0)	1.6 ± 129.5 (0.6–494.3)	3.7 ± 19.0 (1.1–63.9)	6.3 ± 21.1 (1.0–23.6)	0.44
Plasma interleukin-6 (pg/mL)	38.0 ± 38.0 (11.5–168.0)	203.5 ± 1688.0 (8.5–2776.0)	134.5 ± 398.5 (7.5–904.0)	84.0 ± 461.5 (20.5–1952.0)	751.0 ± 1907.0 (12.5–4381.0)	6.5 ± 24.0 (3.0–634.5)	**0.04**
Plasma interleukin-8 (pg/mL)	28.9 ± 12.6 (5.2–94.0)	14.3 ± 31.9 (11.3–66.6)	22.6 ± 8.1 (9.7–68.1)	34.2 ± 25.1 (8.3 ± 107.3)	30.0 ± 12.5 (7.0–36.3)	26.5 ± 21.7 (11.9–44.0)	0.41
Plasma interleukin-10 (pg/mL)	20.9 ± 10.6 (2.6–26.2)	8.5 ± 18.5 (2.6–39.6)	35.7 ± 17.5 (2.6 ± 86.3)	21.9 ± 90.1 (5.0–135.1)	4.4 ± 160.7 (2.6–634.7)	18.0 ± 36.3 (2.6–70.3)	0.64
Plasma interleukin-12 (pg/mL)	4.0 ± 3.5 (3.0–93.8)	3.0 ± 4.3 (3.0–57.8)	3.0 ± 0.1 (3.0–71.9)	3.0 ± 0.9 (3.0–247.8)	3.0 ± 15.2 (3.0–63.7)	3.0 ± 0.3 (3.0–32.2)	0.11
Plasma interleukin-13 (pg/mL)	32.2 ± 62.2 (6.4–274.5)	20.7 ± 100.2 (6.4–264.1)	18.5 ± 76.5 (6.4–111.1)	10.6 ± 109.7 (6.4–408.7)	30.5 ± 68.8 (6.4 ± 137.2)	19.7 ± 30.5 (6.4–79.9)	0.59
Plasma interleukin-15 (pg/mL)	13.8 ± 7.5 (6.9–21.6)	9.7 ± 8.2 (7.9–26.3)	13.5 ± 6.9 (6.4–25.6)	7.6 ± 3.3 (5.3–32.9)	4.3 ± 1.0 (3.0–5.9)	7.2 ± 5.9 (4.0 ± 12.9)	0.68
Plasma interleukin-17A (pg/mL)	6.5 ± 5.9 (1.3–11.1)	4.1 ± 3.8 (1.3–9.2)	4.7 ± 2.8 (1.3–7.4)	1.3 ± 1.4 (1.3–8.3)	1.3 ± 0.1 (1.3–1.3)	1.3 ± 0.1 (1.3–1.3)	**<0.01**
Serum IP-10 (pg/mL)	1732.8 ± 2939.5 (205.0–5024.9)	120.7 ± 587.7 (101.8–1071.0)	166.3 ± 357.7 (63.9–583.4)	216.3 ± 230.3 (79.9–374.1)	104.9 ± 135.3 (73.6–488.9)	135.5 ± 193.9 (95.0–310.1)	0.5
Serum MCP-1 (pg/mL)	972.8 ± 2068.9 (362.9–4650.5)	761.2 ± 680.3 (255.9–1886.2)	600.5 ± 204.6 (372.0–1484.6)	682.2 ± 123.7 (296.5–4010.7)	526.6 ± 432.9 (337.5–1512.0)	468.3 ± 18.8 (335.4–1408.2)	0.5
Serum MIP-1α (pg/mL)	22.7 ± 17.2 (3.0–43.5)	15.1 ± 6.6 (3.0–47.9)	15.1 ± 11.8 (3.0–47.9)	13.1 ± 39.0 (3.0–87.5)	9.8 ± 31.4 (3.0–87.9)	11.3 ± 10.6 (3.0–33.8)	0.4
Serum RANTES (pg/mL)	2416.4 ± 2479.9 (831.1–6169.0)	4172.3 ± 1419.0 (1125.9–17271.7)	3523.7 ± 1976.1 (320.7–7093.2)	2576.8 ± 1186.9 (624.5–3021.3)	2040.4 ± 589.2 (1068.8–2959.3)	1834.2 ± 1262.6 (619.7–2887.7)	0.85
Serum TNF-α (pg/mL)	59.9 ± 44.0 (24.7–155.6)	38.5 ± 33.3 (18.5–80.2)	39.6 ± 16.8 (28.8–60.2)	39.6 ± 152.4 (6.4–244.0)	32.0 ± 37.3 (13.4–100.3)	26.3 ± 18.7 (8.0–47.6)	0.86
Serum TNF-β (pg/mL)	22.0 ± 54.2 (2.2–69.2)	18.5 ± 35.4 (1.6–71.0)	10.8 ± 13.0 (1.6–39.7)	5.3 ± 56.7 (1.6–72.7)	9.2 ± 15.8 (1.6–25.8)	4.6 ± 11.6 (1.6–15.3)	0.33
Serum IgG (g/L)	7.8 ± 3.8 (1.3–15.4)	8.0 ± 1.9 (4.8–12.0)	7.7 ± 0.7 (6.7–8.0)	6.1 ± 1.8 (4.9–11.9)	7.6 ± 0.1 (7.0–9.5)	7.6 ± 2.4 (7.2–9.8)	0.05
Serum IgA (g/L)	1.9 ± 0.9 (0.5–5.1)	2.1 ± 0.8 (0.3–3.3)	1.4 ± 1.0 (0.5–2.6)	2.0 ± 1.8 (0.4–3.0)	1.9 ± 0.1 (1.9–1.9)	1.9 ± 0.7 (0.5–2.0)	0.97
Serum IgM (g/L)	1.3 ± 1.1 (0.1–2.4)	0.8 ± 0.6 (0–1.9)	0.8 ± 1.7 (0–3.5)	1.0 ± 1.2 (0–2.6)	0.9 ± 0.1 (0–0.9)	0.9 ± 0.1 (0.1–3.1)	0.9
Serum electrophoresis/immunofixation	Normal/negative	Normal/negative	Normal/negative	Normal/negative	Normal/negative	Normal/negative	n.a.
Serum C3 (g/L)	1.3 ± 0.3 (0.6–1.7)	1.1 ± 0.3 (1.0–1.6)	1.3 ± 0.8 (0.9–2.0)	1.7 ± 0.4 (0.9–1.7)	0.9 ± 0.1 (0.8–1.0)	1.0 ± 0.1 (0.8–1.8)	1
Serum C4 (g/L)	0.3 ± 0.1 (0.2–0.5)	0.3 ± 0.1 (0.1–0.4)	0.3 ± 0.2 (0–0.4)	0.3 ± 0.3 (0–0.4)	0.1 ± 0.1 (0.1–0.2)	0.2 ± 0.1 (0.1–0.5)	0.97
Serum ENA ELISA	Below c/o	Below c/o	Below c/o	Below c/o	Below c/o	Below c/o	n.a.
Serum anti-dsDNA (IU/mL)	4.0 ± 7.5 (1.0–16.0)	3.0 ± 0.8 (1.0–4.0)	2.5 ± 3.0 (1.0–10.0)	4.0 ± 1.0 (3.0–5.0)	6.5 ± 0.5 (6.0–7.0)	3.0 ± 4.0 (1.0–7.0)	0.46
Serum ANCA IF	Below c/o	Below c/o	Below c/o	Below c/o	Below c/o	Below c/o	n.a.
Serum ANA IF	Below c/o	Below c/o	Below c/o	Below c/o	Below c/o	Below c/o	n.a.

* *p* values are calculated between data measured at inclusion and +28 days *post*-ECP. ANA: antinuclear antibody, ANCA: anti-neutrophil cytoplasmic antibody, C3: complement component 3, C4: complement component 4, c/o: cutoff, dsDNA: double-stranded doxyribonucleic acid, ELISA: enzyme-linked immunosorbent assay, ENA: extractable nuclear antigens, IF: immunofluorescence, IgA: immunoglobulin A, IgG: immunoglobulin G, IgM: immunoglobulin M, n.a.: not applicable.

**Table 5:** Peripheral whole-blood FACS parameters of enrolled patients at baseline, inclusion and post-ECP follow-up.

Parameter	Baseline	Inclusion	*Post*-ECP Follow-Up				*p* Value *
			+3 Days	+7 Days	+14 Days	+28 Days	
**Regulatory T-cell ratio (%)**	0.1 ± 0.1 (0.1–0.3)	0.1 ± 0.1 (0–0.3)	0.4 ± 0.1 (0.4–0.5)	0.1 ± 0.5 (0.1–1.0)	0.3 ± 0.1 (0.3–0.3)	0.2 ± 0.3 (0.1–0.3)	0.74
**CD3+ T-cell count (cells/μL)**	656 ± 654 (277–2377)	367 ± 473 (321–1267)	469 ± 113 (382–609)	802 ± 560 (445–1990)	1987 ± 447 (1536–2430)	1888 ± 789 (1454–4324)	0.28
**CD3+/CD8+ T-cell count (cells/μL)**	215 ± 164 (158–630)	117 ± 71 (97–318)	193 ± 47 (119–213)	194 ± 31 (150–246)	189 ± 77 (89–244)	287 ± 125 (112–361)	0.87
**CD3+/CD4+ T-cell count (cells/μL)**	406 ± 542 (213–1834)	203 ± 677 (96–2187)	481 ± 796 (342–1934)	711 ± 741 (282–1766)	1286 ± 889 (397–2175)	1159 ± 1946 (61–3954)	0.46
**CD3-/CD16+/CD56+ NK-cell count (cells/μL)**	90 ± 33 (65–162)	38 ± 77 (11–245)	56 ± 48 (26–124)	42 ± 24 (20–68)	35 ± 5 (29–39)	59 ± 25 (33–84)	0.88
**CD19+ B-cell count (cells/μL)**	126 ± 69 (59–198)	44 ± 97 (6.0–191.7)	136 ± 213 (96–523)	188 ± 47 (107–202)	235 ± 111 (43–267)	212 ± 197 (59–453)	0.2
**Double-negative T-cell ratio (%)**	1.0 ± 0.7 (0–3.4)	1.7 ± 2.1 (0.2–7.5)	0.8 ± 2.5 (0.6–5.6)	1.1 ± 0.8 (0.5–2.0)	1.1 ± 0.7 (0.4–1.9)	0.4 ± 2.0 (0.2–4.1)	0.28
**Double-positive T-cell ratio (%)**	0.1 ± 0.3 (0.01–0.8)	0.1 ± 0.4 (0.04–1.2)	0.1 ± 0.1 (0.03–0.2)	0.1 ± 0.1 (0.01–0.2)	0.1 ± 0.1 (0.05–0.12)	0.2 ± 0.1 (0.1–0.2)	1.0
**Counts within the CD3+ gate:**							
**CD3+/TCRαβ+ T-cell (cells/μL)**	949 ± 221 (687–1546)	601 ± 631 (212–2368)	489 ± 83 (435–601)	906 ± 774 (434–1982)	567 ± 1128 (164–2421)	1440 ± 2067 (172–4301)	1.0
**CD3+/TCRγδ+ T-cell (cells/μL)**	23.5 ± 19.6 (14.3–53.7)	43.1 ± 37.3 (7.1–77.3)	27.6 ± 21.9 (6.7–45.6)	10.2 ± 2.5 (6.0–11.0)	14.1 ± 4.3 (9.7–18.4)	13.1 ± 3.1 (11.0–17.3)	0.23
**CD3+/CD25+ T-cell (cells/μL)**	54.3 ± 26.5 (14.2–67.1)	19.3 ± 9.9 (2.8–26.6)	63.5 ± 23.4 (42.1–88.9)	77.9 ± 148.0 (21.9–317.9)	48.0 ± 33.5 (14.6–81.5)	60.5 ± 108.6 (12.7–229.8)	0.14
**CD3+/HLA-DR+ T-cell (cells/μL)**	146.6 ± 211.5 (23.1–524.8)	89.6 ± 203.2 (44.7–615.8)	304.9 ± 297.1 (49.1–682.4)	56.1 ± 333.8 (45.0–712.6)	358.9 ± 307.4 (51.2–666.1)	98.1 ± 375.0 (41.7–1370.8)	0.56
**Counts within the CD4+ gate:**							
**CD4+/CD45RA+ naive T-cell (cells/μL)**	162.3 ± 487.3 (86.2–1548.2)	105.8 ± 599.3 (11.9–2045.4)	152.4 ± 110.1 (29.4–249.6)	335.6 ± 767.6 (102.4–1637.5)	1056.0 ± 869.6 (186.4–1925.6)	547.1 ± 1807.0 (16.2–3630.1)	0.69
**CD4+/CD45RO+ memory T-cell (cells/μL)**	89.9 ± 53.9 (26.5–112.2)	113.4 ± 60.8 (52.0–140.0)	206.1 ± 21.9 (184.2–227.9)	178.2 ± 121.1 (129.0–371.2)	229.4 ± 16.4 (213.0–245.9)	286.7 ± 195.7 (45.3–602.7)	0.2
**Counts within the CD8+ gate:**							
**CD8+/CD45RA+ naive T-cell (cells/μL)**	167.2 ± 34.3 (147.8–216.4)	175.0 ± 171.3 (46.6–506.6)	173.5 ± 186.5 (65.3–438.1)	102.2 ± 21.1 (65.2–107.6)	74.0 ± 24.1 (49.9–98.1)	90.5 ± 29.5 (78.5–136.9)	0.93
**CD8+/CD45RO+ memory T-cell (cells/μL)**	42.5 ± 24.8 (12.8–62.4)	76.2 ± 94.1 (20.9–136.4)	51.2 ± 2.2 (48.7–53.1)	90.0 ± 53.3 (41.6–147.7)	159.5 ± 33.1 (126.4–192.6)	144.4 ± 127.0 (21.6–275.7)	0.03

* *p* values are calculated between data measured at inclusion and +28 days *post*-ECP.

## Data Availability

Anonymized data of patients are available from the corresponding author on reasonable request.

## Supplementary Materials

The following supporting information can be downloaded at: https://www.mdpi.com/article/10.3390/jcm12155000/s1, Figure S1: Study flow chart; Materials S1: FACS plots from MNCs of patients enrolled in the study; Table S1: Schematic diagram for data acquisition at baseline, inclusion and follow-up; Table S2: FACS with early and late apoptosis markers and multi-cytokine analysis from ECP *mononuclear cell* collections (MNC) after ECP sessions 1 (first) to 4 (last) of patients enrolled in the study.

## Abbreviations

ANA	anti-nuclear antibody
ANCA	anti-neutrophil cytoplasmic antibody
ARDS	acute respiratory distress syndrome
C	complement
CD	cluster of differentiation
COVID-19	coronavirus disease 2019
CT	computed tomography
ECP	extracorporeal photopheresis
ENA	extractable nuclear antigen
EOT	end of treatment
FACS	fluorescence-activated cell sorting
FiO_2_	fraction of inspired oxygen
ICU	intensive care unit
IFN	interferon
Ig	immunoglobulin
IL	interleukin
LDH	lactate dehydrogenase
LOS	length of stay
NK	natural killer
MNC	mononuclear cell collection
PaO_2_	partial arterial oxygen tension
RNA	ribonucleic acid
RT-PCR	real-time polymerase chain reaction
SARS-CoV-2	severe acute respiratory syndrome coronavirus 2
