# Supplementary material for: Extracorporeal Photopheresis as a Possible Therapeutic Approach for Adults with Severe and Critical COVID-19 Non-Responsive to Standard Treatment: A Pilot Investigational Study
Source: J Clin Med. 2023 Jul 29;12(15):5000. doi: 10.3390/jcm12155000 (PMC10420323; doi:10.3390/jcm12155000)
Supplement: Supplementary file 1 [file jcm-12-05000-s001.zip › -SUPP-MATERIAL-1-FACS plots of patients enrolled.pdf]

Patient 1 ECP MNC 1

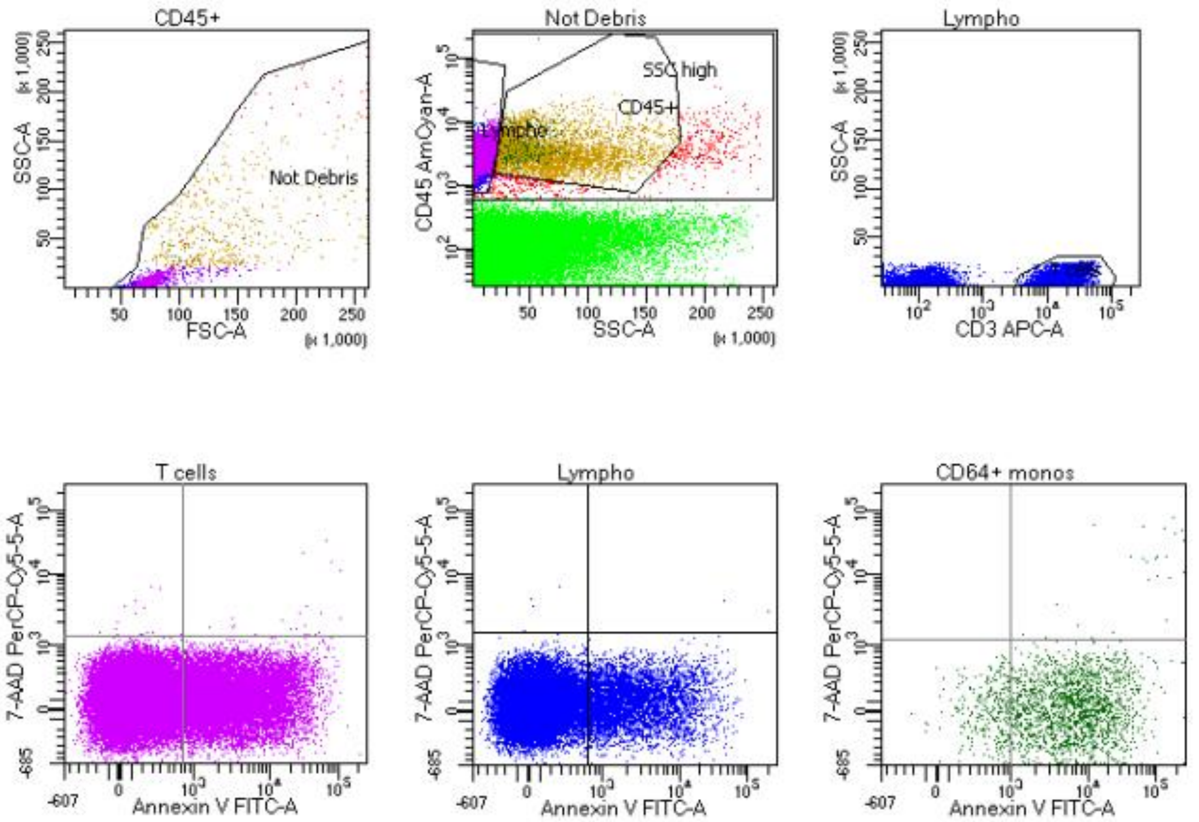

Population	%Parent
⊠ Necrotic - T cells	0.0
⊠ Late apoptotic - T cells	0.0
⊠ Live - T cells	72.1
⊠ Early apoptotic - T cells	27.8

Population	%Parent
⊠ Necrotic - lympho	0.0
⊠ Late apoptotic - lympho	0.0
⊠ Livec - lympho	81.6
⊠ Early apoptotic - lympho	18.4

Population	%P...
⊠ Necrotic - monocytes	0.0
⊠ Late apoptotic - monocytes	1.3
⊠ Live - monocytes	14.8
⊠ Early apoptotic - monocytes	84.0

Tube: annexin/64/7AAD/4/3/8/47/45

Population

- All Events
  - Not Debris
    - CD45+
      - Lympho
        - ⊠ Necrotic - lympho
        - ⊠ Late apoptotic - lympho
        - ⊠ Livec - lympho
        - ⊠ Early apoptotic - lympho
      - T cells
        - ⊠ Necrotic - T cells
        - ⊠ Late apoptotic - T cells
        - ⊠ Live - T cells
        - ⊠ Early apoptotic - T cells
      - SSC high
        - CD64+ monos
          - ⊠ Necrotic - monocytes
          - ⊠ Late apoptotic - monocytes
          - ⊠ Live - monocytes
          - ⊠ Early apoptotic - monocytes

Patient 1 ECP MNC 2

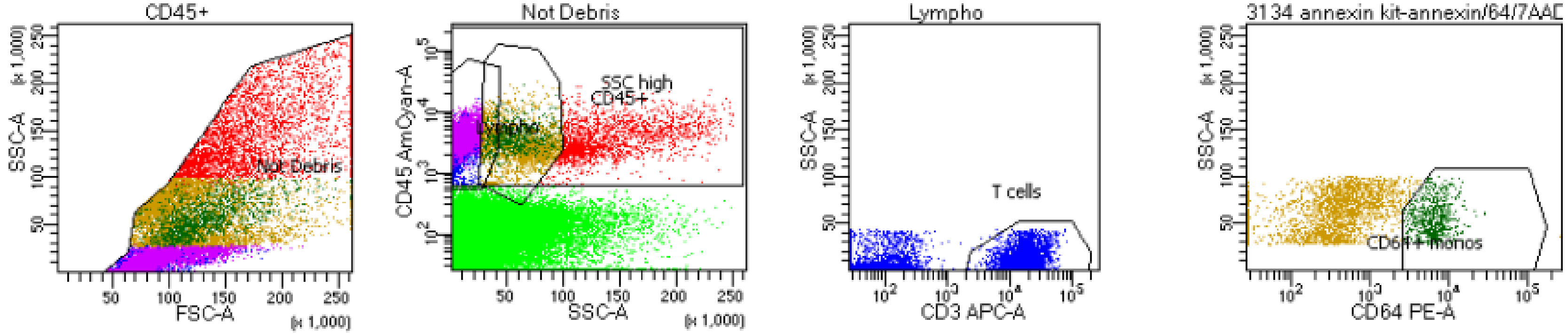

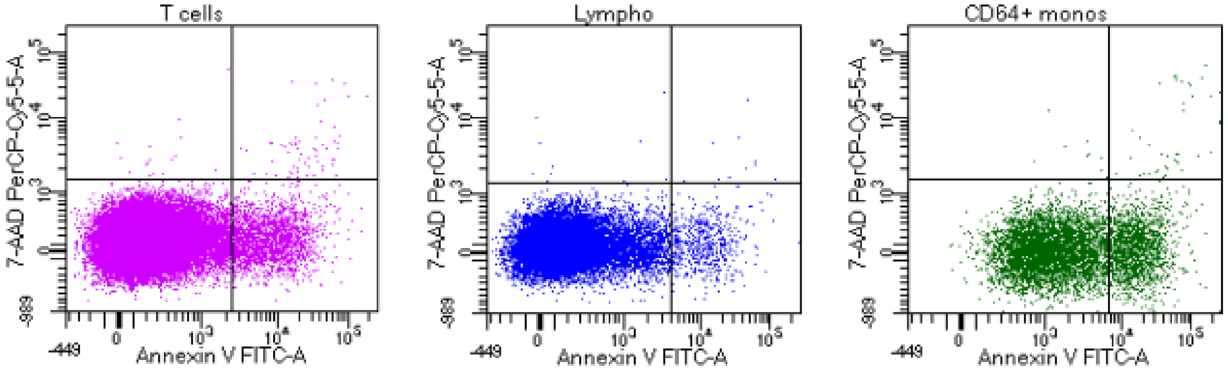

Population	%Parent
Necrotic - T cells	0.0
Late apoptotic - T cells	0.2
Live - T cells	94.5
Early apoptotic - T cells	5.3

Population	%Parent
Necrotic - lympho	0.1
Late apoptotic - lympho	0.1
Livec - lympho	96.9
Early apoptotic - lympho	3.0

Population	%P...
Necrotic - monocytes	0.2
Late apoptotic - monocytes	0.6
Live - monocytes	74.0
Early apoptotic - monocytes	25.2

Tube: annexin/64/7AAD/4/3/8/47/45

Population
- All Events
  - Not Debris
    - CD45+
      - Lympho
        - Necrotic - lympho
        - Late apoptotic - lympho
        - Livec - lympho
        - Early apoptotic - lympho
      - T cells
        - Necrotic - T cells
        - Late apoptotic - T cells
        - Live - T cells
        - Early apoptotic - T cells
      - SSC high
        - CD64+ monos
          - Necrotic - monocytes
          - Late apoptotic - monocytes
          - Live - monocytes
          - Early apoptotic - monocytes

Patient 1 ECP MNC 3

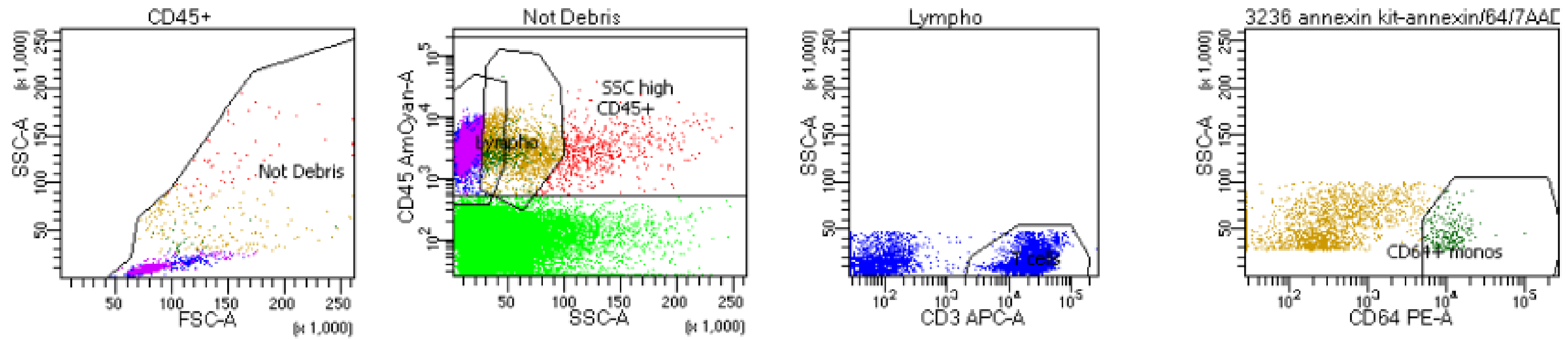

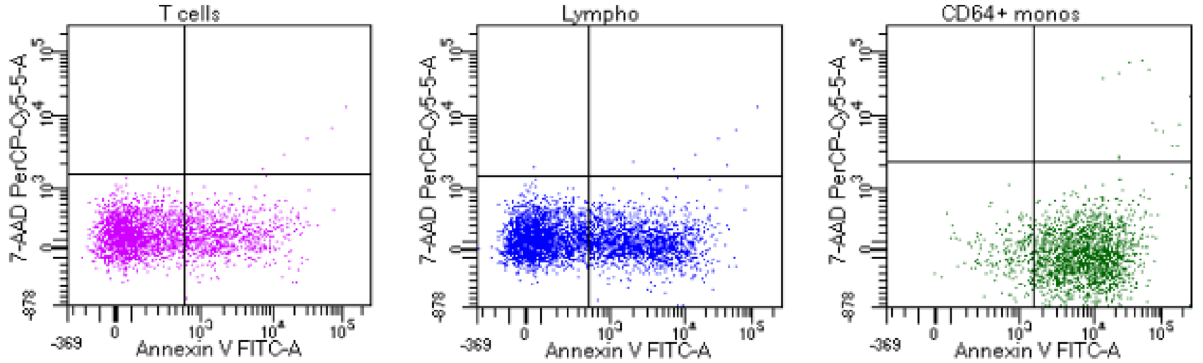

Population	%Parent
Necrotic - T cells	0.0
Late apoptotic - T cells	0.2
Live - T cells	72.0
Early apoptotic - T cells	27.8

Population	%Parent
Necrotic - lympho	0.0
Late apoptotic - lympho	0.2
Livec - lympho	59.5
Early apoptotic - lympho	40.3

Population	%P...
Necrotic - monocytes	0.0
Late apoptotic - monocytes	0.7
Live - monocytes	10.6
Early apoptotic - monocytes	88.8

Tube: annexin/64/7AAD/4/3/8/47/45

Population
- All Events
  - Not Debris
    - CD45+
      - Lympho
        - Necrotic - lympho
        - Late apoptotic - lympho
        - Livec - lympho
        - Early apoptotic - lympho
      - T cells
        - Necrotic - T cells
        - Late apoptotic - T cells
        - Live - T cells
        - Early apoptotic - T cells
      - SSC high
        - CD64+ monos
          - Necrotic - monocytes
          - Late apoptotic - monocytes
          - Live - monocytes
          - Early apoptotic - monocytes

Patient 1 ECP MNC 4

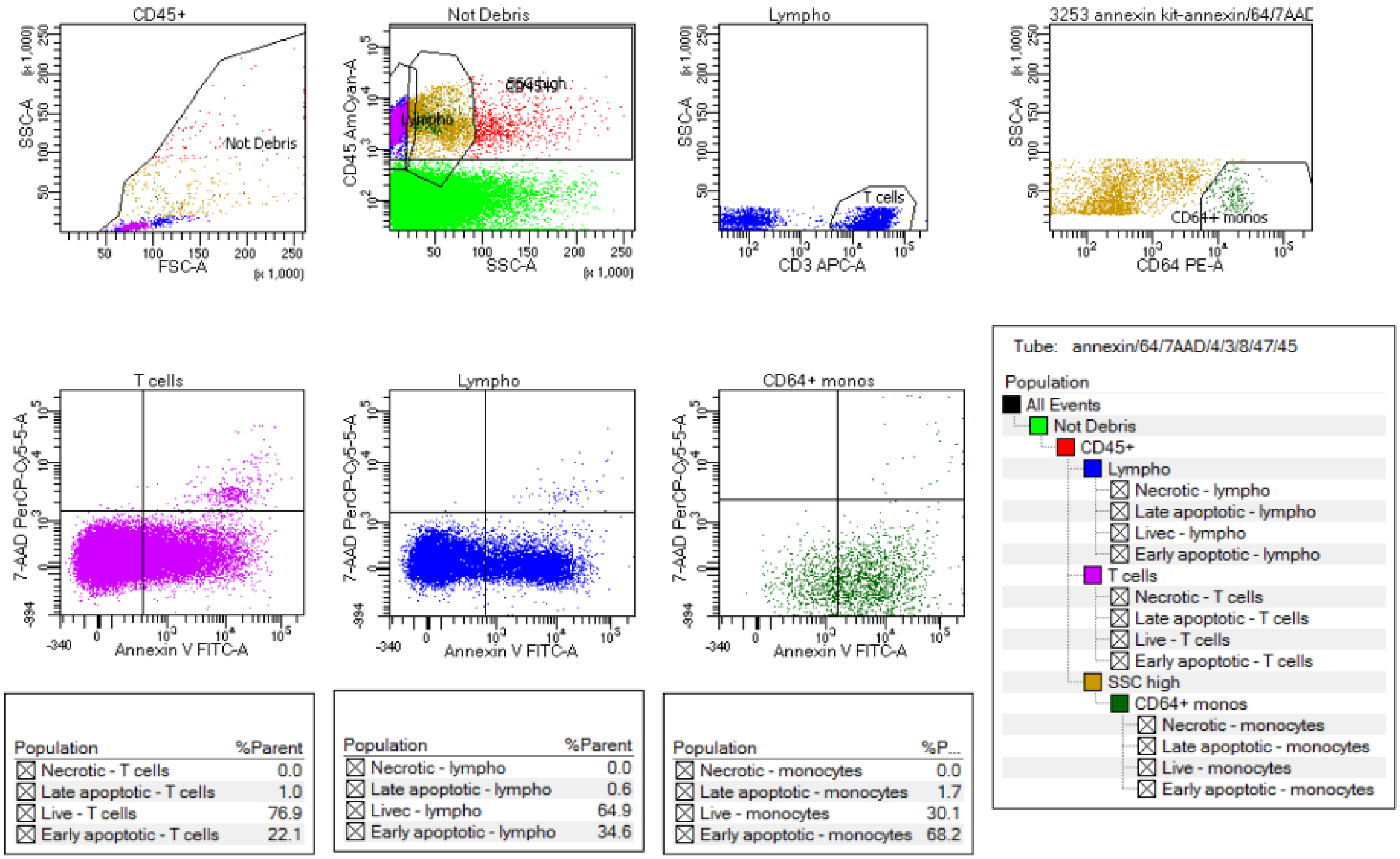

Population	%Parent
Necrotic - T cells	0.0
Late apoptotic - T cells	1.0
Live - T cells	76.9
Early apoptotic - T cells	22.1

Population	%Parent
Necrotic - lympho	0.0
Late apoptotic - lympho	0.6
Livec - lympho	64.9
Early apoptotic - lympho	34.6

Population	%P...
Necrotic - monocytes	0.0
Late apoptotic - monocytes	1.7
Live - monocytes	30.1
Early apoptotic - monocytes	68.2

Tube: annexin/64/7AAD/4/3/8/47/45

Population

- All Events
  - Not Debris
    - CD45+
      - Lympho
        - Necrotic - lympho
        - Late apoptotic - lympho
        - Livec - lympho
        - Early apoptotic - lympho
      - T cells
        - Necrotic - T cells
        - Late apoptotic - T cells
        - Live - T cells
        - Early apoptotic - T cells
      - SSC high
        - CD64+ monos
          - Necrotic - monocytes
          - Late apoptotic - monocytes
          - Live - monocytes
          - Early apoptotic - monocytes

Patient 2 ECP MNC 1

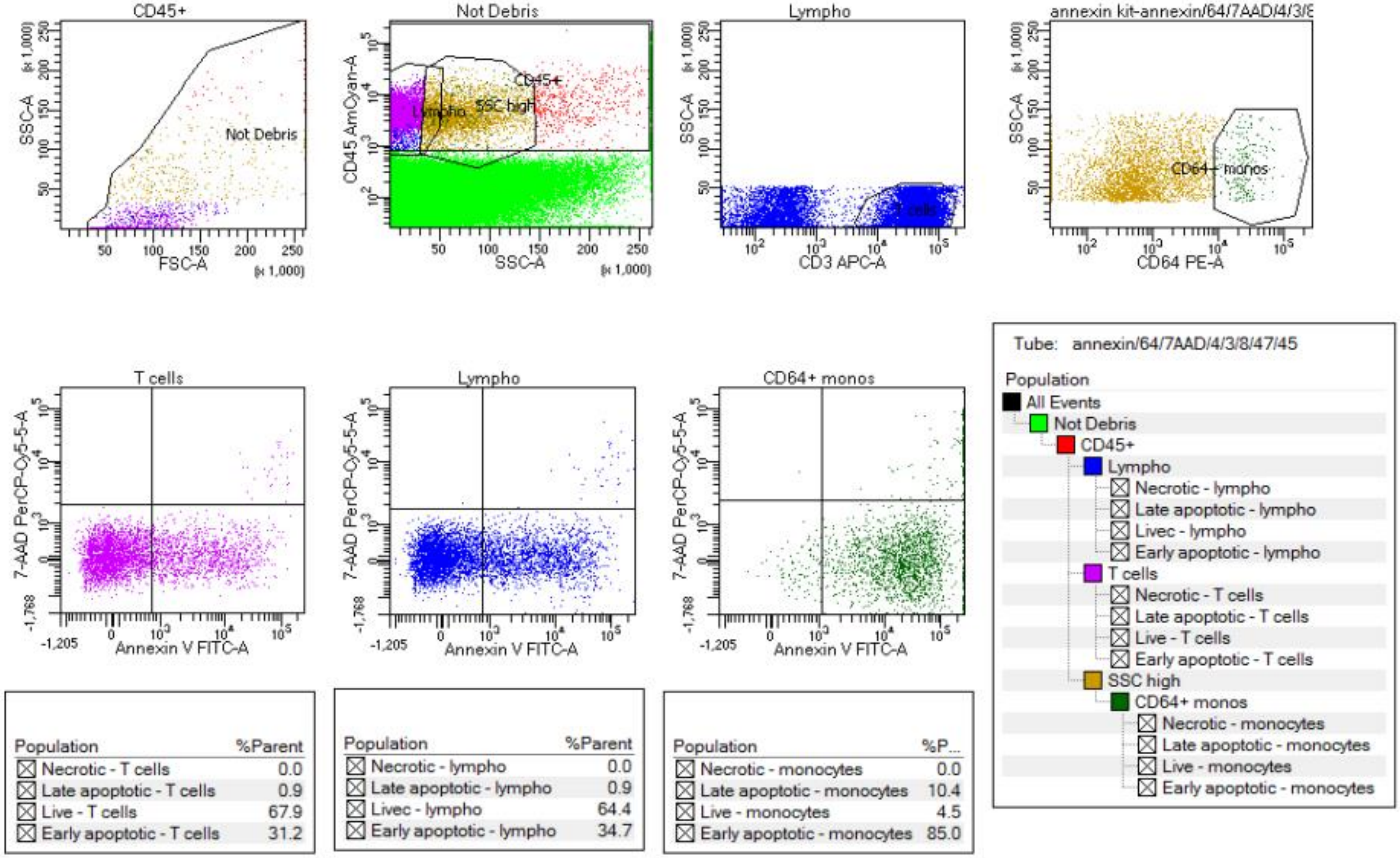

Population	%Parent
Necrotic - T cells	0.0
Late apoptotic - T cells	0.9
Live - T cells	67.9
Early apoptotic - T cells	31.2

Population	%Parent
Necrotic - lympho	0.0
Late apoptotic - lympho	0.9
Livec - lympho	64.4
Early apoptotic - lympho	34.7

Population	%P...
Necrotic - monocytes	0.0
Late apoptotic - monocytes	10.4
Live - monocytes	4.5
Early apoptotic - monocytes	85.0

Patient 2 ECP MNC 2

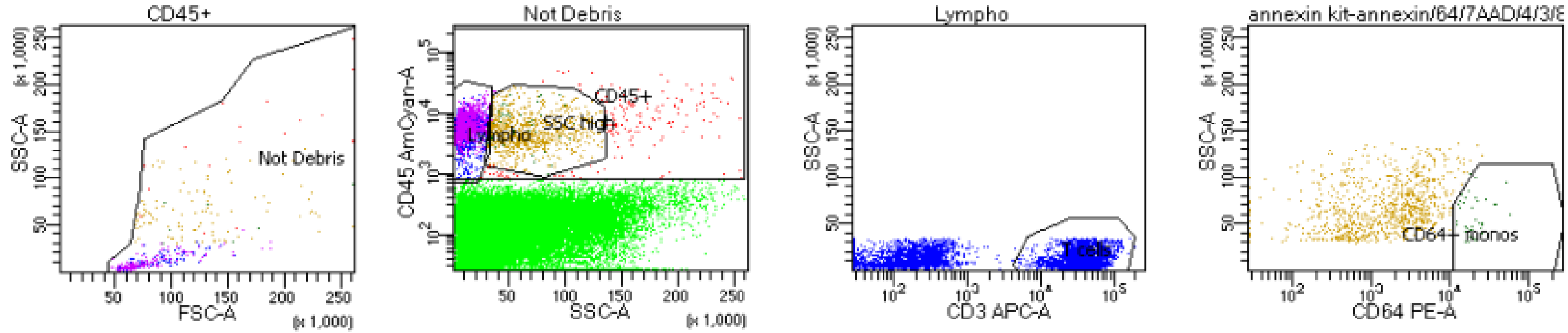

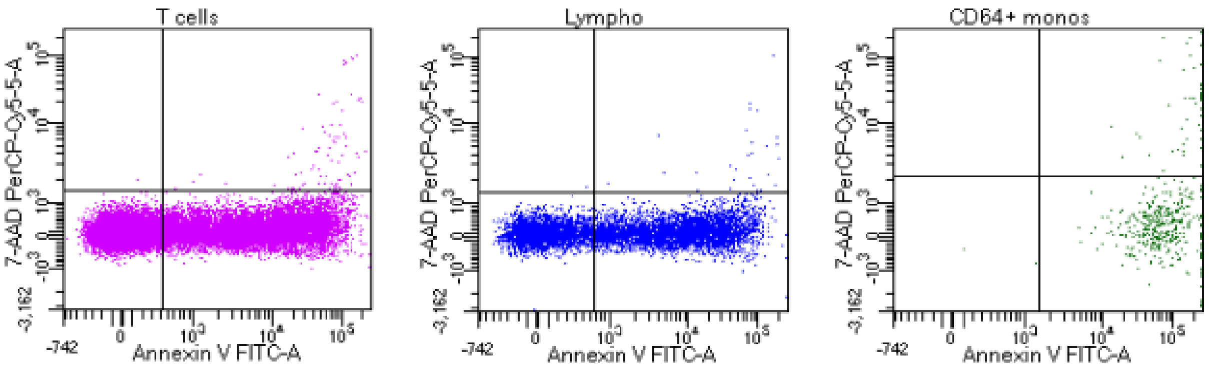

Population	%Parent
Necrotic - T cells	0.0
Late apoptotic - T cells	0.4
Live - T cells	41.4
Early apoptotic - T cells	58.1

Population	%Parent
Necrotic - lympho	0.0
Late apoptotic - lympho	0.4
Livec - lympho	41.4
Early apoptotic - lympho	58.2

Population	%P...
Necrotic - monocytes	0.0
Late apoptotic - monocytes	12.7
Live - monocytes	0.5
Early apoptotic - monocytes	86.8

Tube: annexin/64/7AAD/4/3/8/47/45

Population

- All Events
  - Not Debris
    - CD45+
      - Lympho
        - Necrotic - lympho
        - Late apoptotic - lympho
        - Livec - lympho
        - Early apoptotic - lympho
      - T cells
        - Necrotic - T cells
        - Late apoptotic - T cells
        - Live - T cells
        - Early apoptotic - T cells
      - SSC high
        - CD64+ monos
          - Necrotic - monocytes
          - Late apoptotic - monocytes
          - Live - monocytes
          - Early apoptotic - monocytes

Patient 2 ECP MNC 3

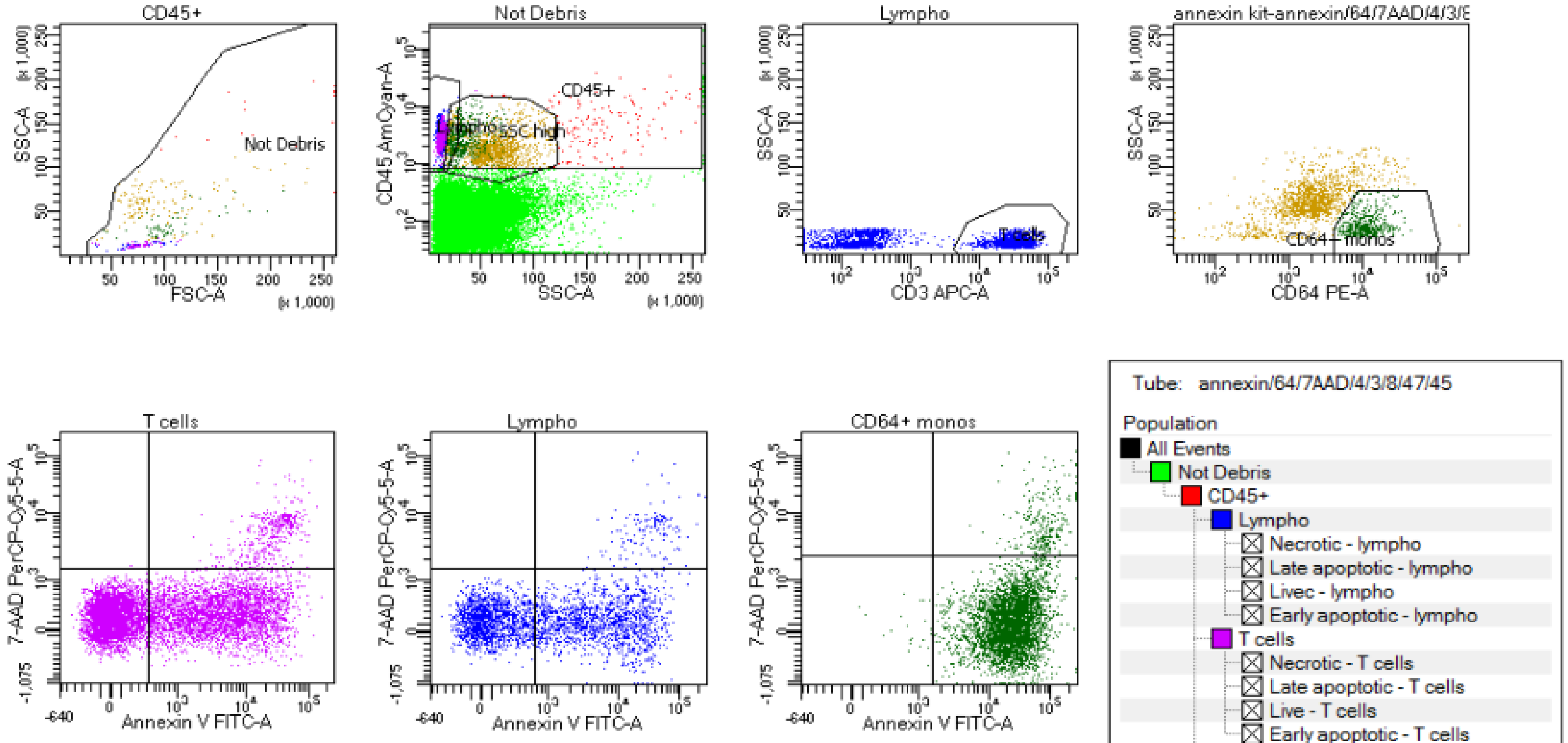

Population	%Parent
Necrotic - T cells	0.0
Late apoptotic - T cells	5.2
Live - T cells	51.5
Early apoptotic - T cells	43.2

Population	%Parent
Necrotic - lympho	0.0
Late apoptotic - lympho	4.3
Livec - lympho	49.1
Early apoptotic - lympho	46.6

Population	%P...
Necrotic - monocytes	0.0
Late apoptotic - monocytes	6.5
Live - monocytes	0.5
Early apoptotic - monocytes	93.0

Patient 2 ECP MNC 4

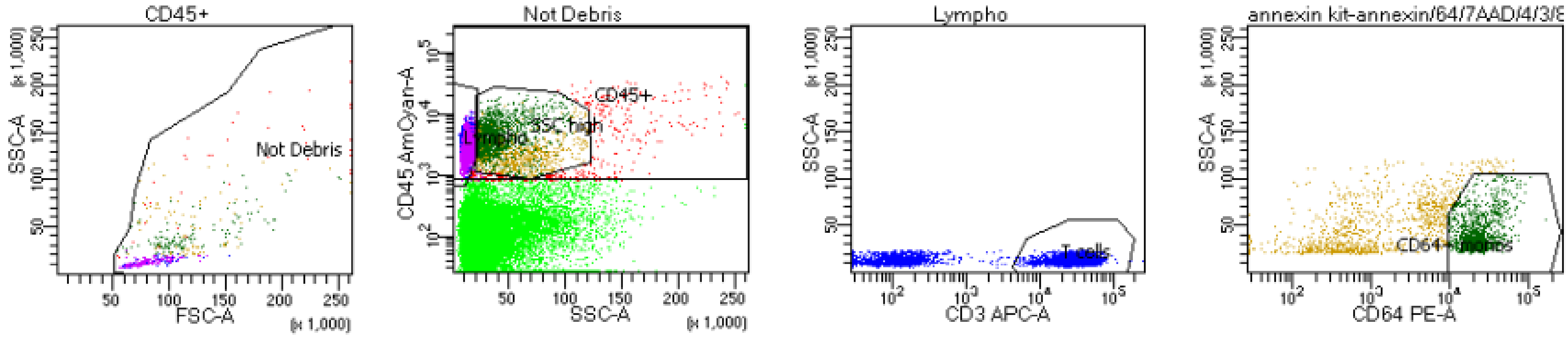

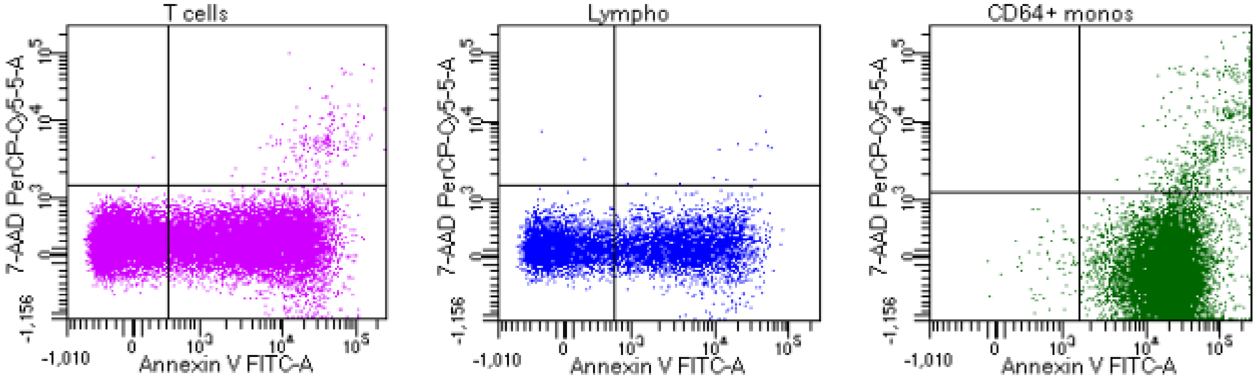

Population	%Parent
Necrotic - T cells	0.0
Late apoptotic - T cells	0.9
Live - T cells	41.7
Early apoptotic - T cells	57.4

Population	%Parent
Necrotic - lympho	0.0
Late apoptotic - lympho	0.3
Livec - lympho	50.0
Early apoptotic - lympho	49.7

Population	%P...
Necrotic - monocytes	0.0
Late apoptotic - monocytes	4.9
Live - monocytes	0.5
Early apoptotic - monocytes	94.6

Tube: annexin/64/7AAD/4/3/8/47/45

Population
- All Events
  - Not Debris
    - CD45+
      - Lympho
        - Necrotic - lympho
        - Late apoptotic - lympho
        - Livec - lympho
        - Early apoptotic - lympho
      - T cells
        - Necrotic - T cells
        - Late apoptotic - T cells
        - Live - T cells
        - Early apoptotic - T cells
      - SSC high
        - CD64+ monos
          - Necrotic - monocytes
          - Late apoptotic - monocytes
          - Live - monocytes
          - Early apoptotic - monocytes

Patient 3 ECP MNC 1

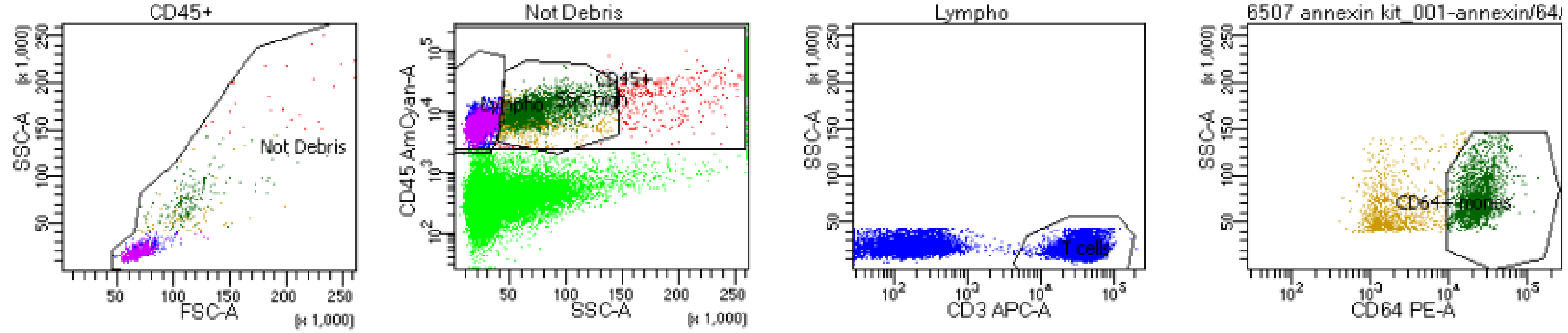

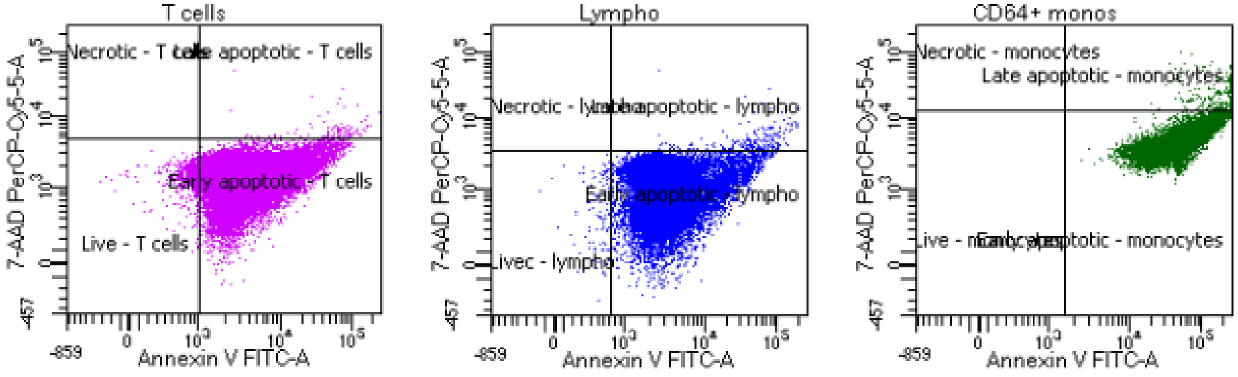

Population	%Parent
Necrotic - T cells	0.0
Late apoptotic - T cells	0.3
Live - T cells	2.1
Early apoptotic - T cells	97.7

Population	%Parent
Necrotic - lympho	0.0
Late apoptotic - lympho	1.3
Livec - lympho	0.6
Early apoptotic - lympho	98.1

Population	%P...
Necrotic - monocytes	0.0
Late apoptotic - monocytes	1.6
Live - monocytes	0.0
Early apoptotic - monocytes	98.4

Tube: annexin/64/7AAD/4/3/8/47/45

Population
- All Events
  - Not Debris
    - CD45+
      - Lympho
        - Necrotic - lympho
        - Late apoptotic - lympho
        - Livec - lympho
        - Early apoptotic - lympho
      - T cells
        - Necrotic - T cells
        - Late apoptotic - T cells
        - Live - T cells
        - Early apoptotic - T cells
      - SSC high
        - CD64+ monos
          - Necrotic - monocytes
          - Late apoptotic - monocytes
          - Live - monocytes
          - Early apoptotic - monocytes

Patient 3 ECP MNC 2

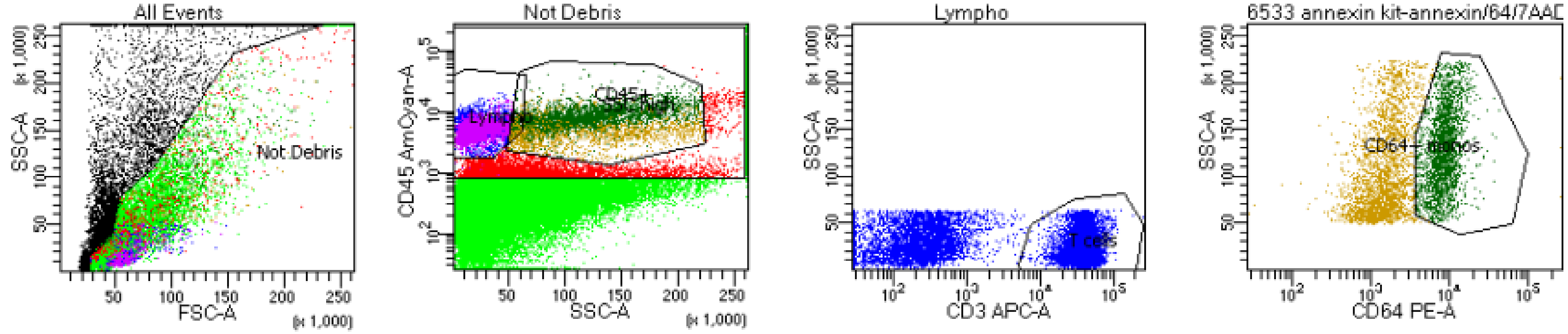

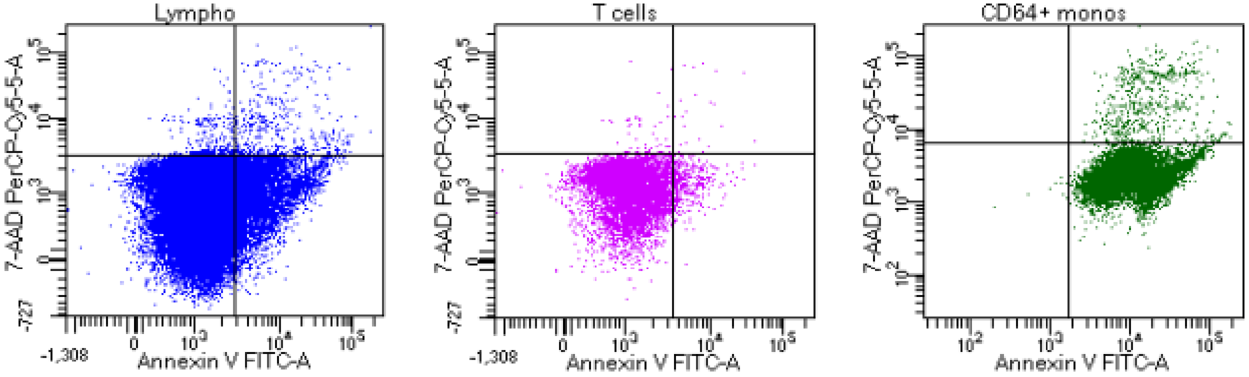

Population	%Parent
Necrotic - T cells	1.0
Late apoptotic - T cells	0.6
Live - T cells	89.2
Early apoptotic - T cells	9.2

Population	%Parent
Necrotic - lympho	0.5
Late apoptotic - lympho	0.6
Livec - lympho	89.1
Early apoptotic - lympho	9.8

Population	%P...
Necrotic - monocytes	0.0
Late apoptotic - monocytes	2.7
Live - monocytes	0.0
Early apoptotic - monocytes	97.2

Tube: annexin/64/7AAD/4/3/8/47/45

Population
- All Events
  - Not Debris
    - CD45+
      - Lympho
        - Necrotic - lympho
        - Late apoptotic - lympho
        - Livec - lympho
        - Early apoptotic - lympho
      - T cells
        - Necrotic - T cells
        - Late apoptotic - T cells
        - Live - T cells
        - Early apoptotic - T cells
      - SSC high
        - CD64+ monos
          - Necrotic - monocytes
          - Late apoptotic - monocytes
          - Live - monocytes
          - Early apoptotic - monocytes

Patient 3 ECP MNC 3

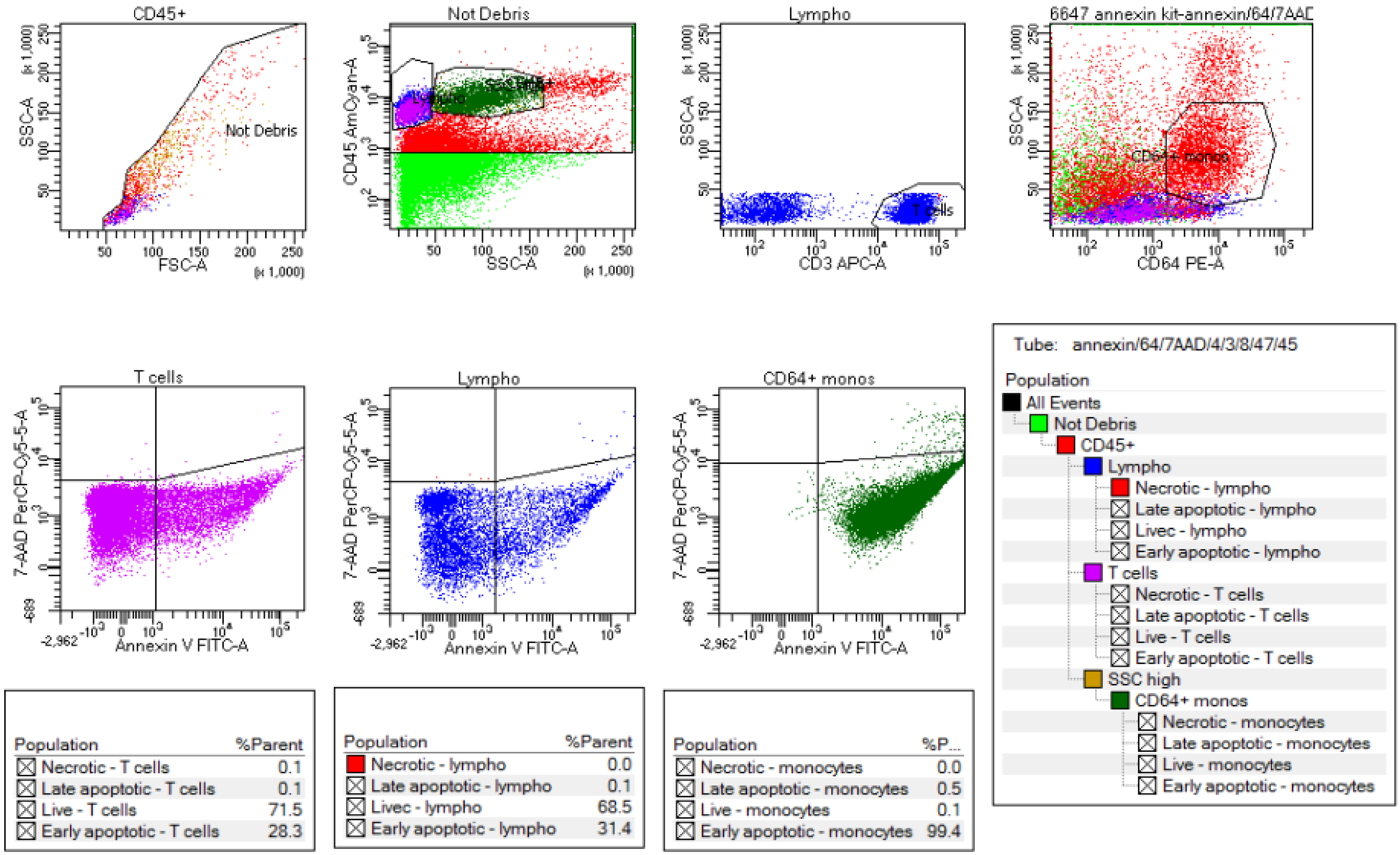

Population	%Parent
Necrotic - T cells	0.1
Late apoptotic - T cells	0.1
Live - T cells	71.5
Early apoptotic - T cells	28.3

Population	%Parent
Necrotic - lympho	0.0
Late apoptotic - lympho	0.1
Livec - lympho	68.5
Early apoptotic - lympho	31.4

Population	%P...
Necrotic - monocytes	0.0
Late apoptotic - monocytes	0.5
Live - monocytes	0.1
Early apoptotic - monocytes	99.4

Tube: annexin/64/7AAD/4/3/8/47/45

Population

- All Events
  - Not Debris
    - CD45+
      - Lympho
        - Necrotic - lympho
        - Late apoptotic - lympho
        - Livec - lympho
        - Early apoptotic - lympho
      - T cells
        - Necrotic - T cells
        - Late apoptotic - T cells
        - Live - T cells
        - Early apoptotic - T cells
      - SSC high
        - CD64+ monos
          - Necrotic - monocytes
          - Late apoptotic - monocytes
          - Live - monocytes
          - Early apoptotic - monocytes

Patient 3 ECP MNC 4

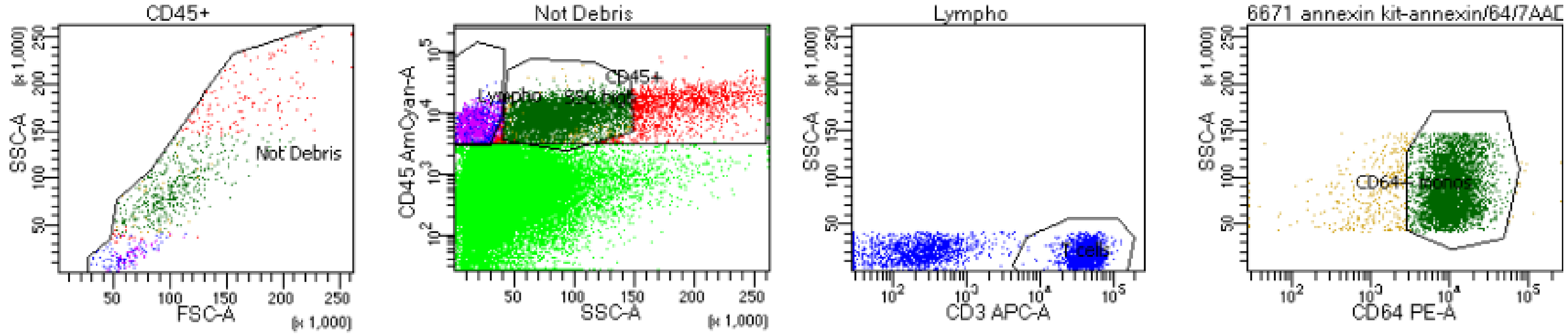

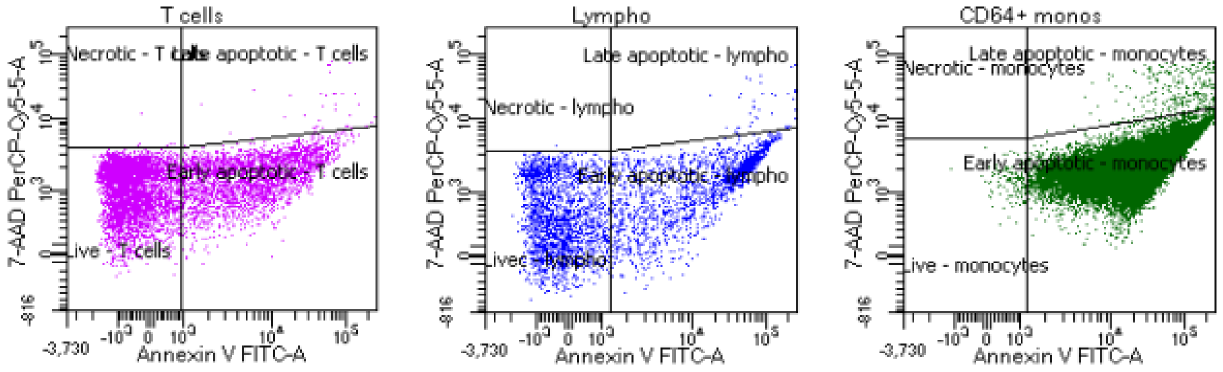

Population	%Parent
Necrotic - T cells	0.1
Late apoptotic - T cells	0.5
Live - T cells	61.7
Early apoptotic - T cells	37.7

Population	%Parent
Necrotic - lympho	0.1
Late apoptotic - lympho	1.2
Livec - lympho	54.5
Early apoptotic - lympho	44.2

Population	%P...
Necrotic - monocytes	0.0
Late apoptotic - monocytes	0.6
Live - monocytes	0.3
Early apoptotic - monocytes	99.0

Tube: annexin/64/7AAD/4/3/8/47/45

Population
- All Events
  - Not Debris
    - CD45+
      - Lympho
        - Necrotic - lympho
        - Late apoptotic - lympho
        - Livec - lympho
        - Early apoptotic - lympho
        - T cells
          - Necrotic - T cells
          - Late apoptotic - T cells
          - Live - T cells
          - Early apoptotic - T cells
      - SSC high
        - CD64+ monos
          - Necrotic - monocytes
          - Late apoptotic - monocytes
          - Live - monocytes
          - Early apoptotic - monocytes

Patient 4 ECP MNC 1

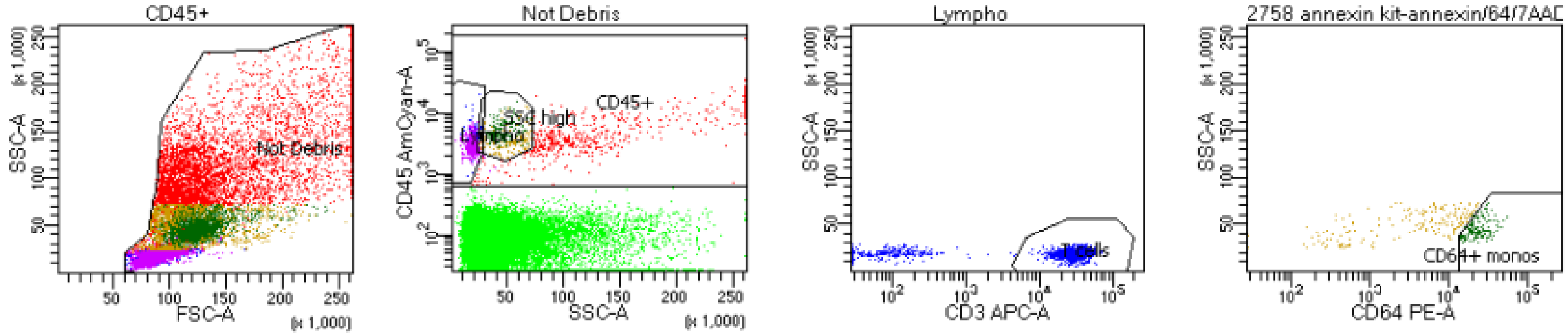

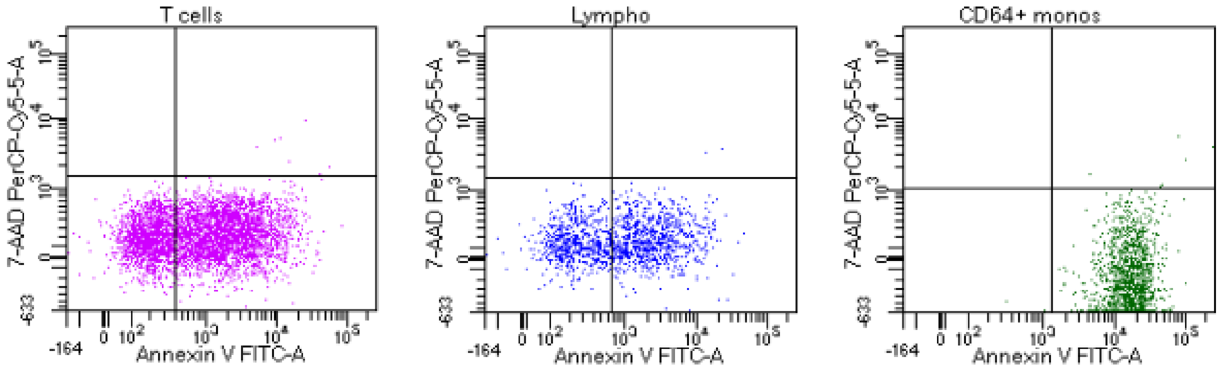

Population	%Parent
Necrotic - T cells	0.0
Late apoptotic - T cells	0.1
Live - T cells	36.3
Early apoptotic - T cells	63.6

Population	%Parent
Necrotic - lympho	0.0
Late apoptotic - lympho	0.2
Livec - lympho	45.0
Early apoptotic - lympho	54.8

Population	%P...
Necrotic - monocytes	0.0
Late apoptotic - monocytes	0.4
Live - monocytes	0.1
Early apoptotic - monocytes	99.5

Tube: annexin/64/7AAD/4/3/8/47/45

Population
- All Events
  - Not Debris
    - CD45+
      - Lympho
        - Necrotic - lympho
        - Late apoptotic - lympho
        - Livec - lympho
        - Early apoptotic - lympho
      - T cells
        - Necrotic - T cells
        - Late apoptotic - T cells
        - Live - T cells
        - Early apoptotic - T cells
      - SSC high
        - CD64+ monos
          - Necrotic - monocytes
          - Late apoptotic - monocytes
          - Live - monocytes
          - Early apoptotic - monocytes

Patient 4 ECP MNC 2

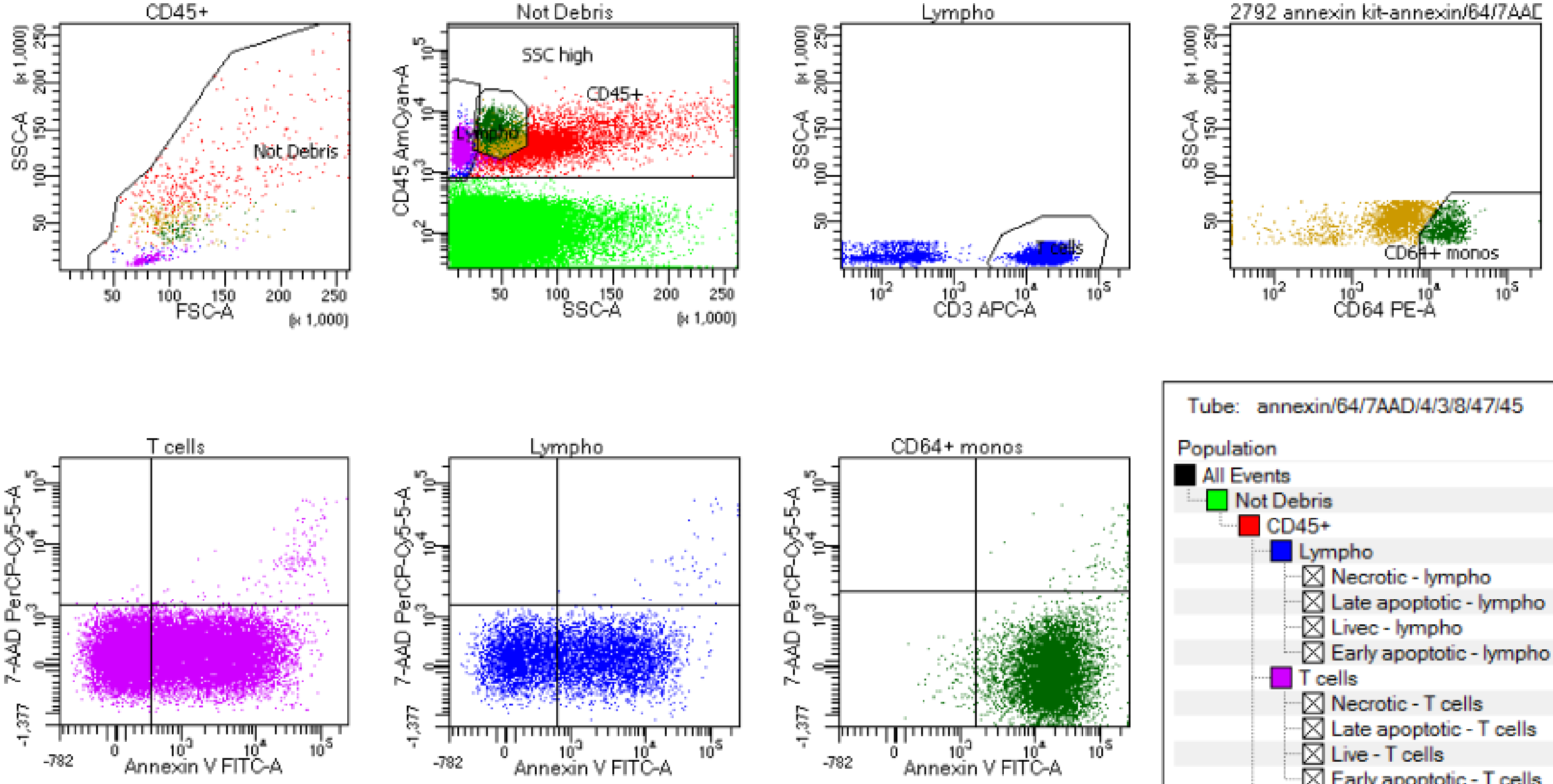

Population	%Parent
Necrotic - T cells	0.1
Late apoptotic - T cells	0.8
Live - T cells	38.6
Early apoptotic - T cells	60.6

Population	%Parent
Necrotic - lympho	0.0
Late apoptotic - lympho	0.7
Livec - lympho	44.9
Early apoptotic - lympho	54.4

Population	%P...
Necrotic - monocytes	0.0
Late apoptotic - monocytes	1.1
Live - monocytes	1.1
Early apoptotic - monocytes	97.9

Tube: annexin/64/7AAD/4/3/8/47/45

Population

- All Events
  - Not Debris
    - CD45+
      - Lympho
        - Necrotic - lympho
        - Late apoptotic - lympho
        - Livec - lympho
        - Early apoptotic - lympho
      - T cells
        - Necrotic - T cells
        - Late apoptotic - T cells
        - Live - T cells
        - Early apoptotic - T cells
      - SSC high
        - CD64+ monos
          - Necrotic - monocytes
          - Late apoptotic - monocytes
          - Live - monocytes
          - Early apoptotic - monocytes

Patient 4 ECP MNC 3

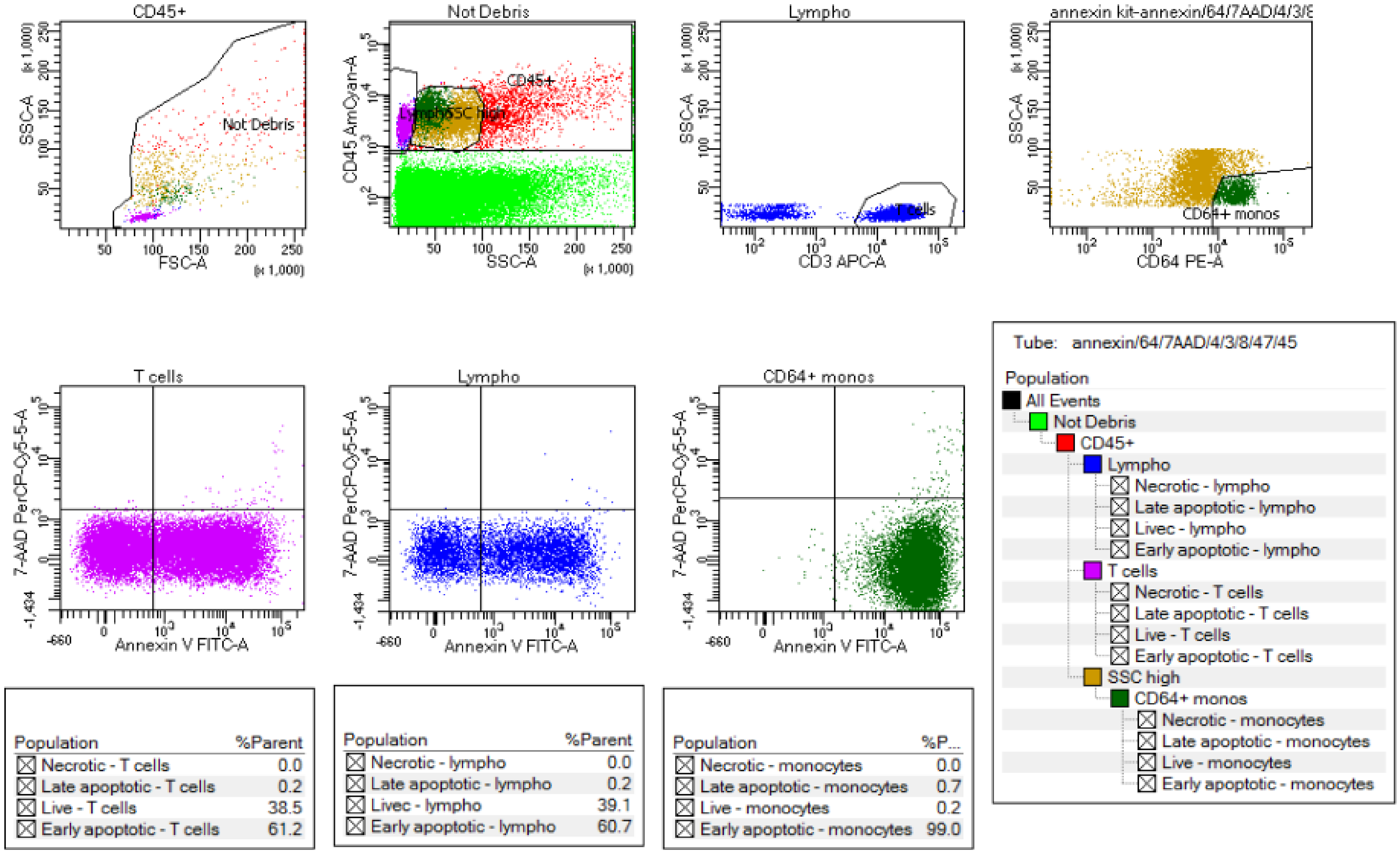

Population	%Parent
Necrotic - T cells	0.0
Late apoptotic - T cells	0.2
Live - T cells	38.5
Early apoptotic - T cells	61.2

Population	%Parent
Necrotic - lympho	0.0
Late apoptotic - lympho	0.2
Livec - lympho	39.1
Early apoptotic - lympho	60.7

Population	%P...
Necrotic - monocytes	0.0
Late apoptotic - monocytes	0.7
Live - monocytes	0.2
Early apoptotic - monocytes	99.0

Tube: annexin/64/7AAD/4/3/8/47/45

Population

- All Events
  - Not Debris
    - CD45+
      - Lympho
        - Necrotic - lympho
        - Late apoptotic - lympho
        - Livec - lympho
        - Early apoptotic - lympho
      - T cells
        - Necrotic - T cells
        - Late apoptotic - T cells
        - Live - T cells
        - Early apoptotic - T cells
      - SSC high
        - CD64+ monos
          - Necrotic - monocytes
          - Late apoptotic - monocytes
          - Live - monocytes
          - Early apoptotic - monocytes

Patient 4 ECP MNC 4

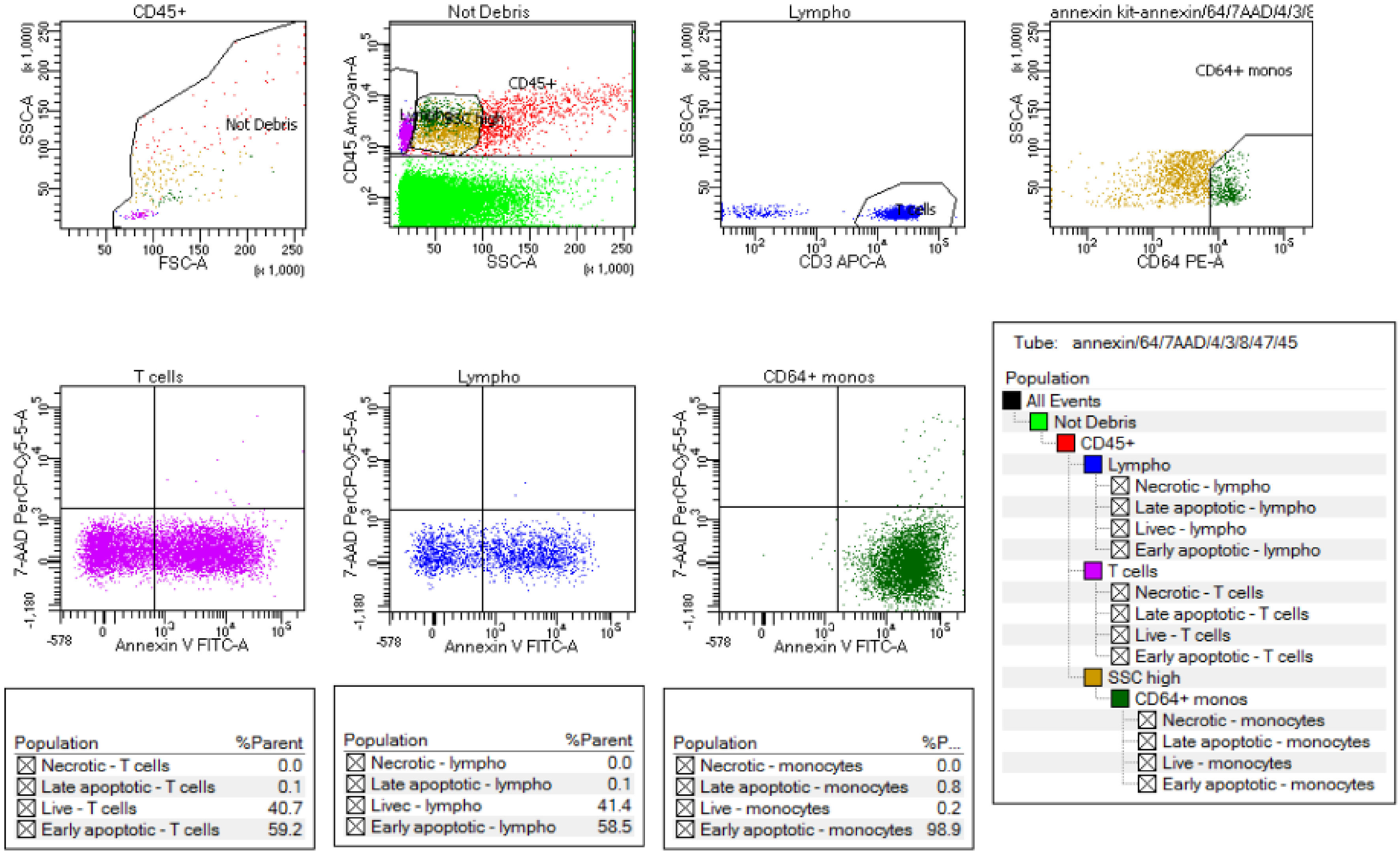

Population	%Parent
Necrotic - T cells	0.0
Late apoptotic - T cells	0.1
Live - T cells	40.7
Early apoptotic - T cells	59.2

Population	%Parent
Necrotic - lympho	0.0
Late apoptotic - lympho	0.1
Livec - lympho	41.4
Early apoptotic - lympho	58.5

Population	%P...
Necrotic - monocytes	0.0
Late apoptotic - monocytes	0.8
Live - monocytes	0.2
Early apoptotic - monocytes	98.9

Tube: annexin/64/7AAD/4/3/8/47/45

Population
- All Events
  - Not Debris
    - CD45+
      - Lympho
        - Necrotic - lympho
        - Late apoptotic - lympho
        - Livec - lympho
        - Early apoptotic - lympho
      - T cells
        - Necrotic - T cells
        - Late apoptotic - T cells
        - Live - T cells
        - Early apoptotic - T cells
      - SSC high
        - CD64+ monos
          - Necrotic - monocytes
          - Late apoptotic - monocytes
          - Live - monocytes
          - Early apoptotic - monocytes

Patient 5 ECP MNC 1

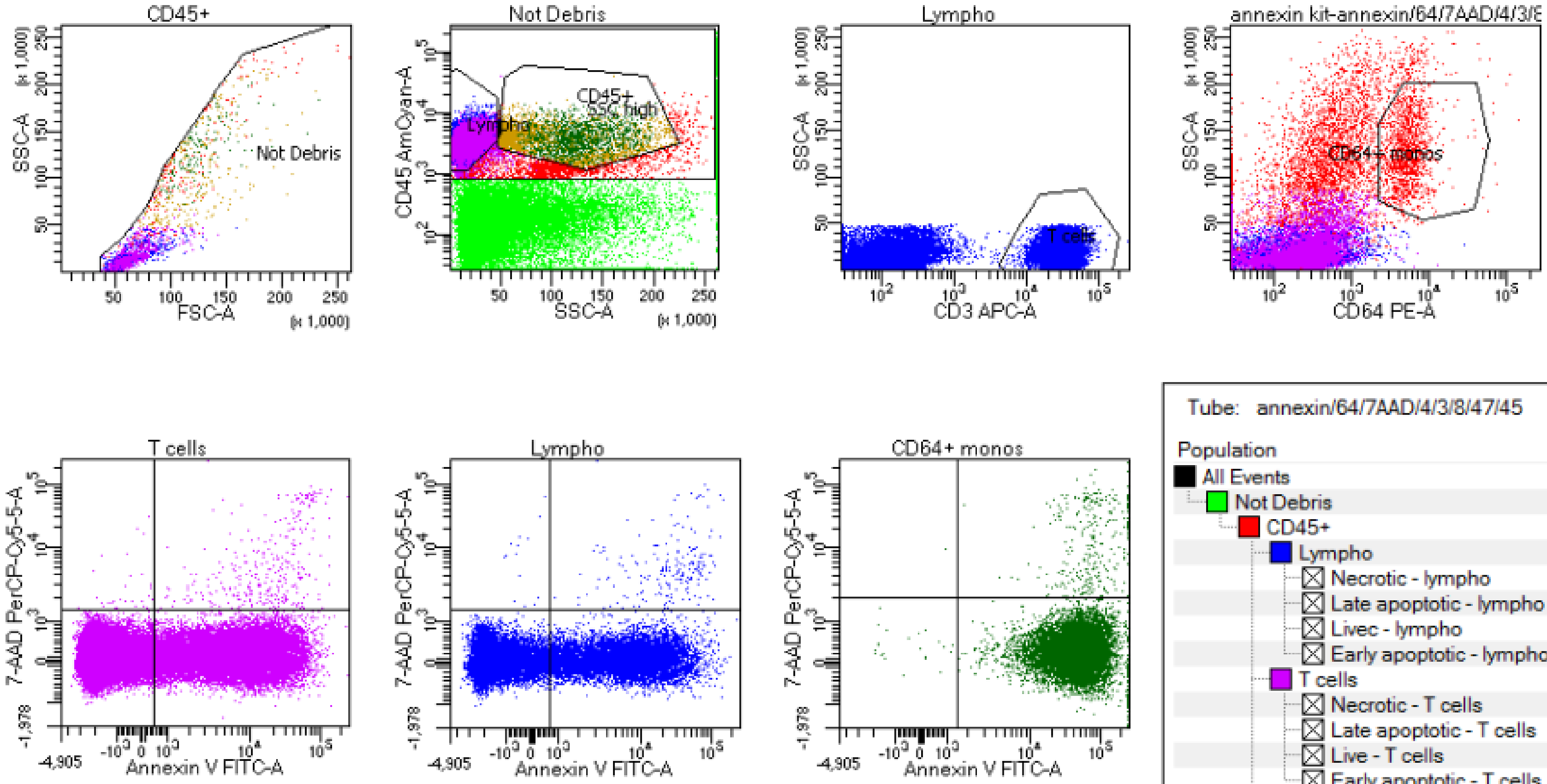

Population	%Parent
Necrotic - T cells	0.0
Late apoptotic - T cells	0.5
Live - T cells	67.6
Early apoptotic - T cells	31.9

Population	%Parent
Necrotic - lympho	0.0
Late apoptotic - lympho	0.7
Livec - lympho	68.0
Early apoptotic - lympho	31.3

Population	%P...
Necrotic - monocytes	0.0
Late apoptotic - monocytes	2.4
Live - monocytes	0.2
Early apoptotic - monocytes	97.3

Tube: annexin/64/7AAD/4/3/8/47/45

Population
- All Events
  - Not Debris
    - CD45+
      - Lympho
        - Necrotic - lympho
        - Late apoptotic - lympho
        - Livec - lympho
        - Early apoptotic - lympho
      - T cells
        - Necrotic - T cells
        - Late apoptotic - T cells
        - Live - T cells
        - Early apoptotic - T cells
      - SSC high
        - CD64+ monos
          - Necrotic - monocytes
          - Late apoptotic - monocytes
          - Live - monocytes
          - Early apoptotic - monocytes

Patient 5 ECP MNC 2

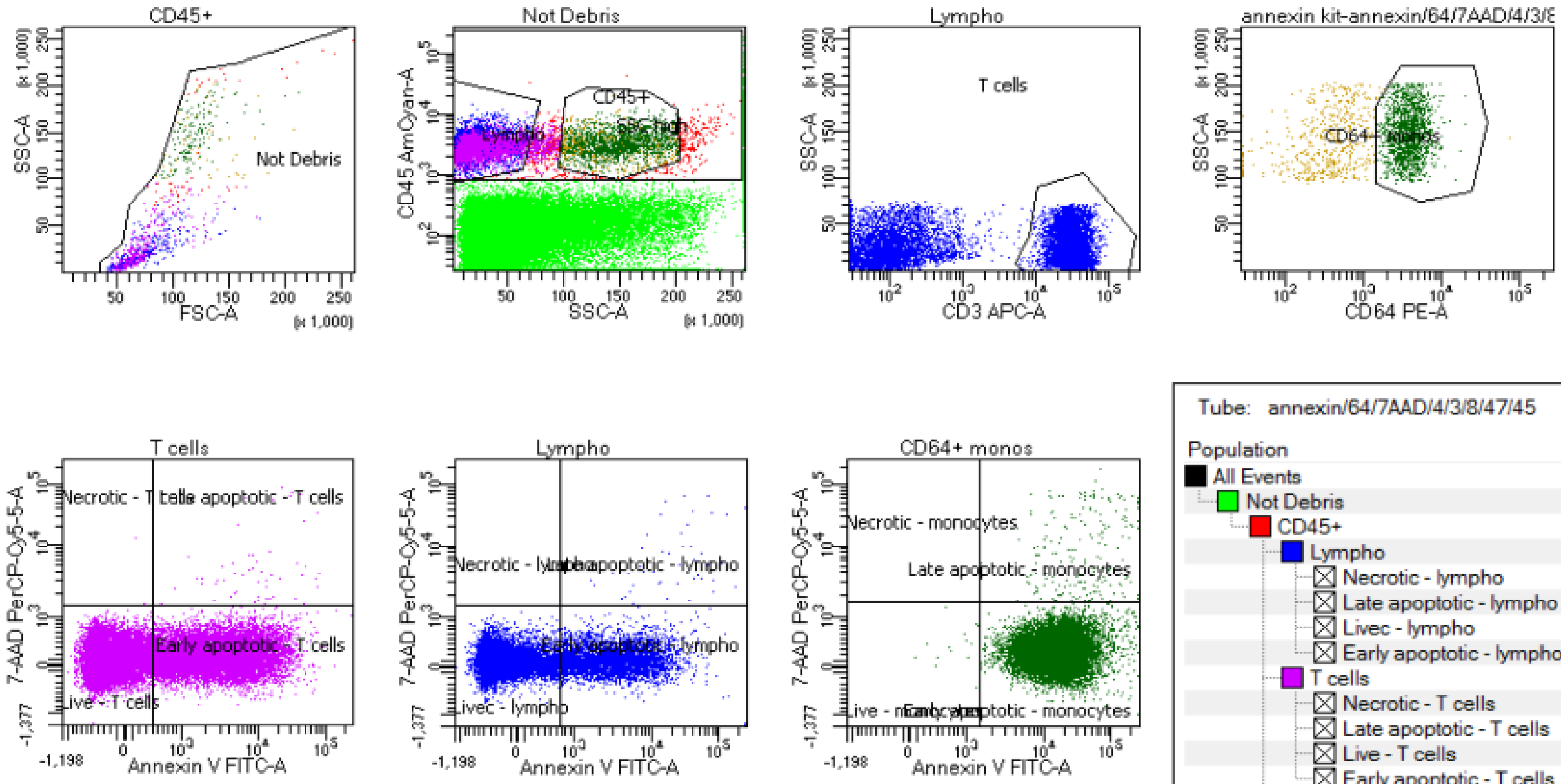

Population	%Parent
Necrotic - T cells	0.0
Late apoptotic - T cells	0.2
Live - T cells	60.8
Early apoptotic - T cells	39.0

Population	%Parent
Necrotic - lympho	0.0
Late apoptotic - lympho	0.3
Livec - lympho	68.2
Early apoptotic - lympho	31.5

Population	%P...
Necrotic - monocytes	0.0
Late apoptotic - monocytes	1.4
Live - monocytes	0.1
Early apoptotic - monocytes	98.5

Tube: annexin/64/7AAD/4/3/8/47/45

Population
- All Events
  - Not Debris
    - CD45+
      - Lympho
        - Necrotic - lympho
        - Late apoptotic - lympho
        - Livec - lympho
        - Early apoptotic - lympho
        - T cells
          - Necrotic - T cells
          - Late apoptotic - T cells
          - Live - T cells
          - Early apoptotic - T cells
      - SSC high
        - CD64+ monos
          - Necrotic - monocytes
          - Late apoptotic - monocytes
          - Live - monocytes
          - Early apoptotic - monocytes

Patient 5 ECP MNC 3

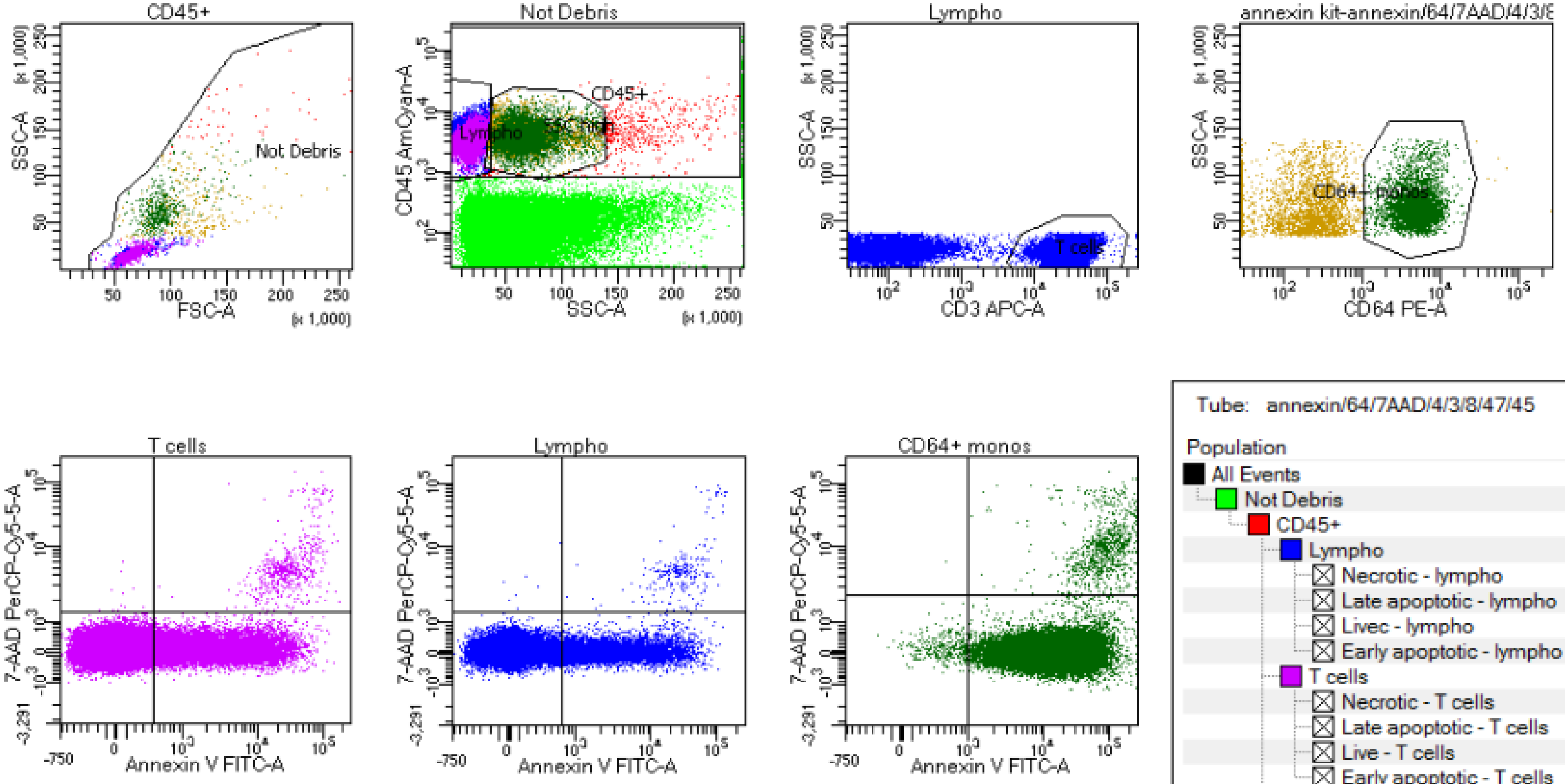

Population	%Parent
Necrotic - T cells	0.0
Late apoptotic - T cells	0.6
Live - T cells	83.4
Early apoptotic - T cells	16.0

Population	%Parent
Necrotic - lympho	0.0
Late apoptotic - lympho	0.6
Livec - lympho	79.8
Early apoptotic - lympho	19.6

Population	%P...
Necrotic - monocytes	0.0
Late apoptotic - monocytes	2.2
Live - monocytes	0.9
Early apoptotic - monocytes	96.9

Tube: annexin/64/7AAD/4/3/8/47/45

Population
- All Events
  - Not Debris
    - CD45+
      - Lympho
        - Necrotic - lympho
        - Late apoptotic - lympho
        - Livec - lympho
        - Early apoptotic - lympho
      - T cells
        - Necrotic - T cells
        - Late apoptotic - T cells
        - Live - T cells
        - Early apoptotic - T cells
      - SSC high
        - CD64+ monos
          - Necrotic - monocytes
          - Late apoptotic - monocytes
          - Live - monocytes
          - Early apoptotic - monocytes

Patient 5 ECP MNC 4

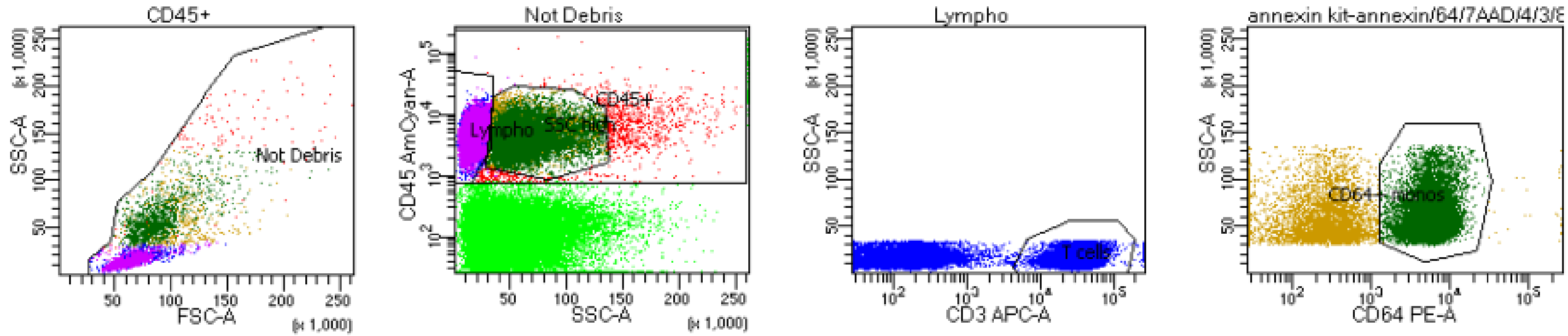

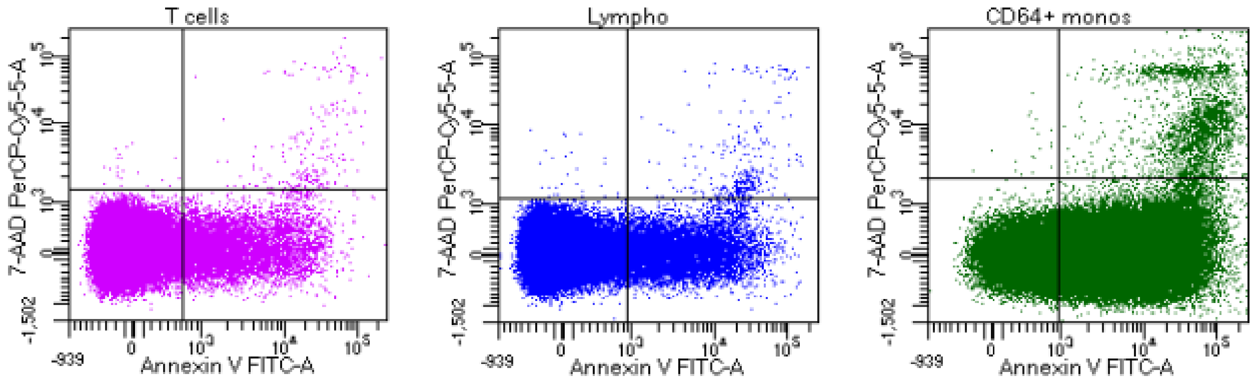

Population	%Parent
Necrotic - T cells	0.0
Late apoptotic - T cells	0.3
Live - T cells	87.9
Early apoptotic - T cells	11.8

Population	%Parent
Necrotic - lympho	0.1
Late apoptotic - lympho	0.5
Livec - lympho	86.6
Early apoptotic - lympho	12.9

Population	%P...
Necrotic - monocytes	0.0
Late apoptotic - monocytes	1.8
Live - monocytes	16.9
Early apoptotic - monocytes	81.3

Tube: annexin/64/7AAD/4/3/8/47/45

Population

- All Events
  - Not Debris
    - CD45+
      - Lympho
        - Necrotic - lympho
        - Late apoptotic - lympho
        - Livec - lympho
        - Early apoptotic - lympho
      - T cells
        - Necrotic - T cells
        - Late apoptotic - T cells
        - Live - T cells
        - Early apoptotic - T cells
      - SSC high
        - CD64+ monos
          - Necrotic - monocytes
          - Late apoptotic - monocytes
          - Live - monocytes
          - Early apoptotic - monocytes

Patient 6 ECP MNC 1

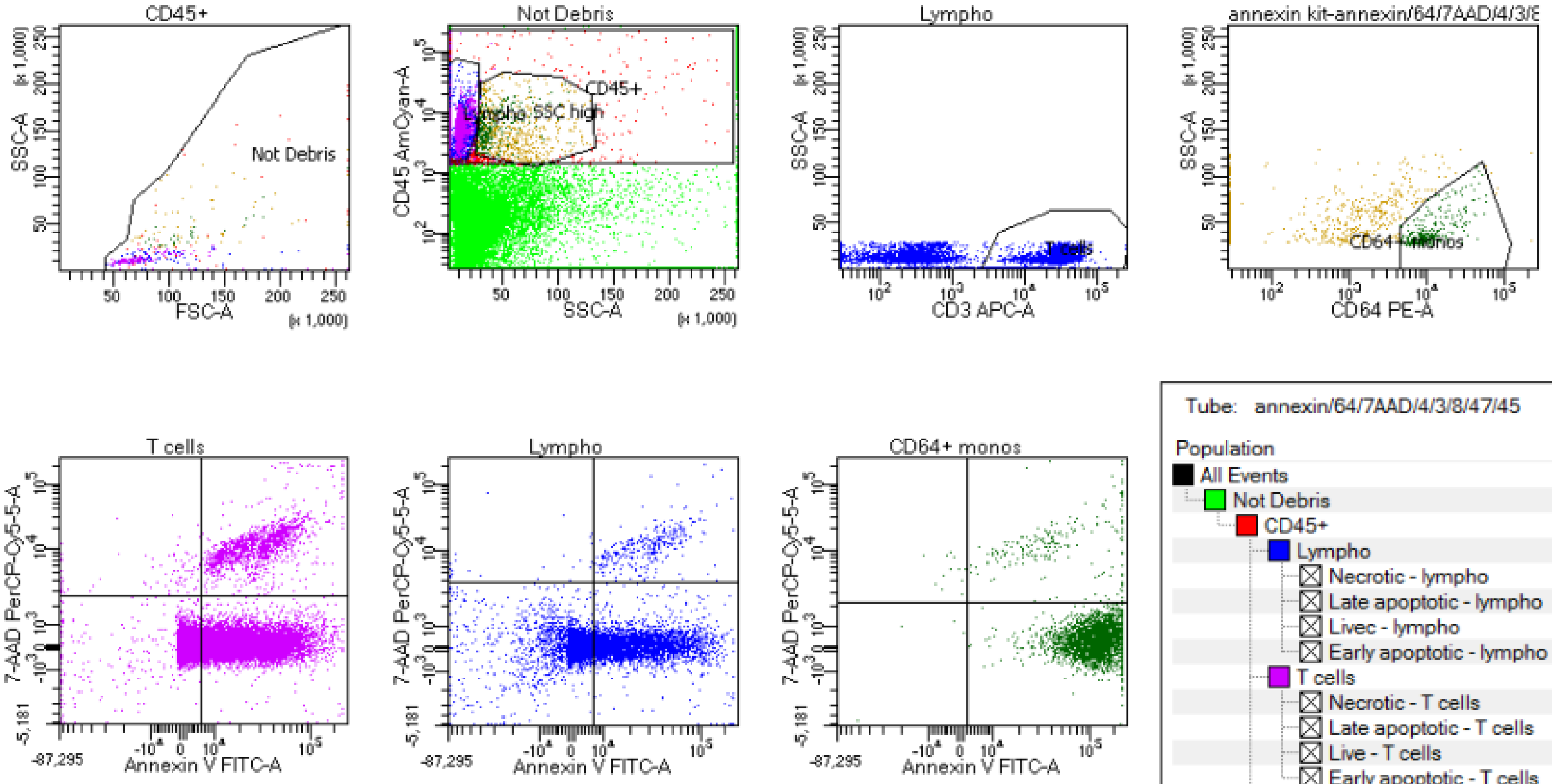

Population	%Parent
Necrotic - T cells	0.5
Late apoptotic - T cells	8.0
Live - T cells	39.2
Early apoptotic - T cells	52.3

Population	%Parent
Necrotic - lympho	0.4
Late apoptotic - lympho	4.5
Livec - lympho	52.2
Early apoptotic - lympho	42.9

Population	%P...
Necrotic - monocytes	0.4
Late apoptotic - monocytes	7.3
Live - monocytes	0.1
Early apoptotic - monocytes	92.1

Tube: annexin/64/7AAD/4/3/8/47/45

Population

- All Events
  - Not Debris
    - CD45+
      - Lympho
        - Necrotic - lympho
        - Late apoptotic - lympho
        - Livec - lympho
        - Early apoptotic - lympho
      - T cells
        - Necrotic - T cells
        - Late apoptotic - T cells
        - Live - T cells
        - Early apoptotic - T cells
      - SSC high
        - CD64+ monos
          - Necrotic - monocytes
          - Late apoptotic - monocytes
          - Live - monocytes
          - Early apoptotic - monocytes

Patient 6 ECP MNC 2

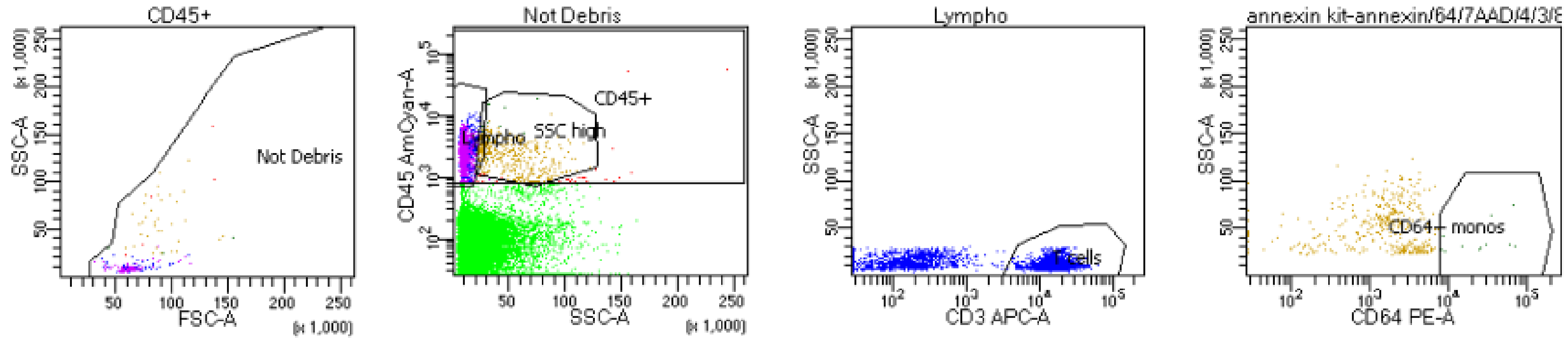

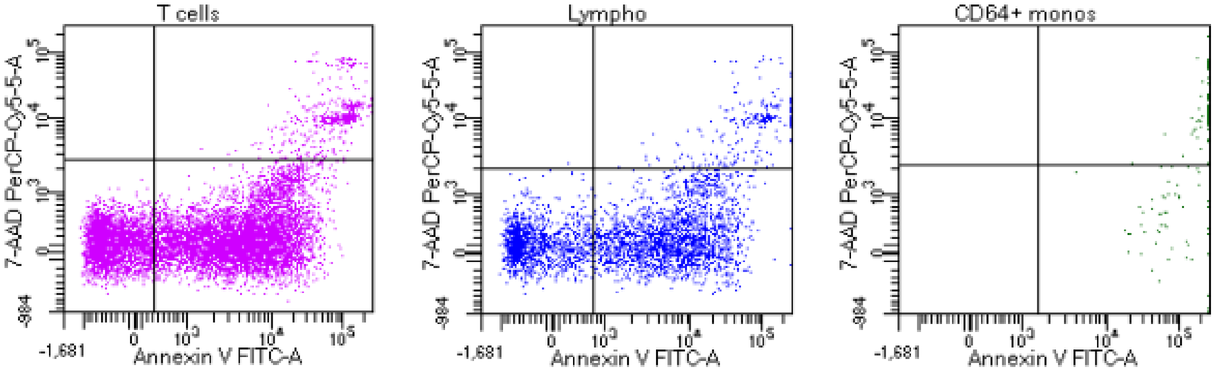

Population	%Parent
Necrotic - T cells	0.0
Late apoptotic - T cells	5.3
Live - T cells	31.6
Early apoptotic - T cells	63.2

Population	%Parent
Necrotic - lympho	0.0
Late apoptotic - lympho	6.8
Livec - lympho	35.5
Early apoptotic - lympho	57.6

Population	%P...
Necrotic - monocytes	0.0
Late apoptotic - monocytes	59.9
Live - monocytes	0.0
Early apoptotic - monocytes	40.1

Tube: annexin/64/7AAD/4/3/8/47/45

Population
- All Events
  - Not Debris
    - CD45+
      - Lympho
        - Necrotic - lympho
        - Late apoptotic - lympho
        - Livec - lympho
        - Early apoptotic - lympho
      - T cells
        - Necrotic - T cells
        - Late apoptotic - T cells
        - Live - T cells
        - Early apoptotic - T cells
      - SSC high
        - CD64+ monos
          - Necrotic - monocytes
          - Late apoptotic - monocytes
          - Live - monocytes
          - Early apoptotic - monocytes

Patient 6 ECP MNC 3

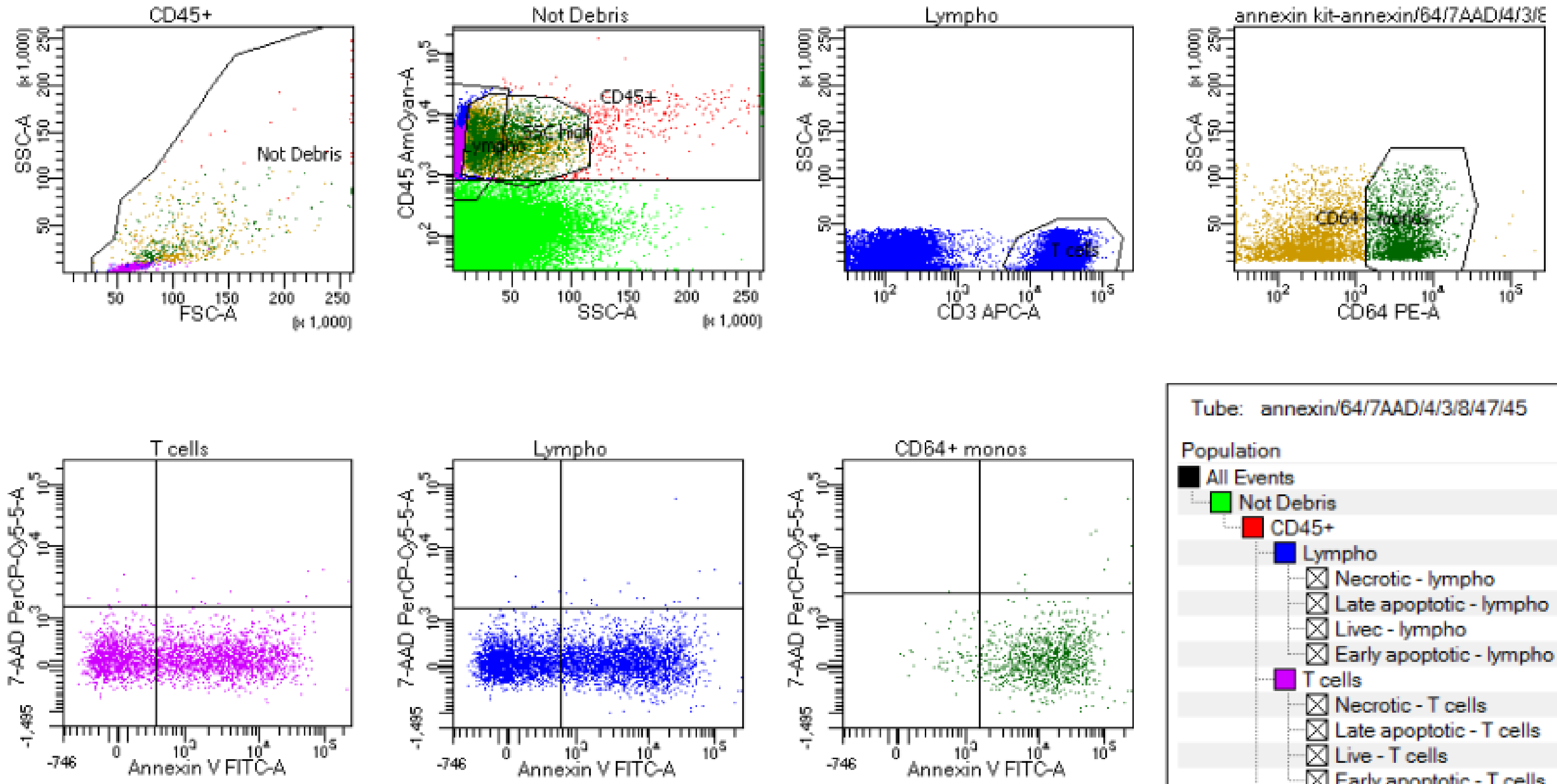

Population	%Parent
Necrotic - T cells	0.2
Late apoptotic - T cells	0.5
Live - T cells	42.4
Early apoptotic - T cells	56.9

Population	%Parent
Necrotic - lympho	0.2
Late apoptotic - lympho	0.5
Livec - lympho	45.6
Early apoptotic - lympho	53.7

Population	%P...
Necrotic - monocytes	0.0
Late apoptotic - monocytes	1.1
Live - monocytes	6.3
Early apoptotic - monocytes	92.5

Tube: annexin/64/7AAD/4/3/8/47/45

Population
- All Events
  - Not Debris
    - CD45+
      - Lympho
        - Necrotic - lympho
        - Late apoptotic - lympho
        - Livec - lympho
        - Early apoptotic - lympho
      - T cells
        - Necrotic - T cells
        - Late apoptotic - T cells
        - Live - T cells
        - Early apoptotic - T cells
      - SSC high
        - CD64+ monos
          - Necrotic - monocytes
          - Late apoptotic - monocytes
          - Live - monocytes
          - Early apoptotic - monocytes

Patient 6 ECP MNC 4

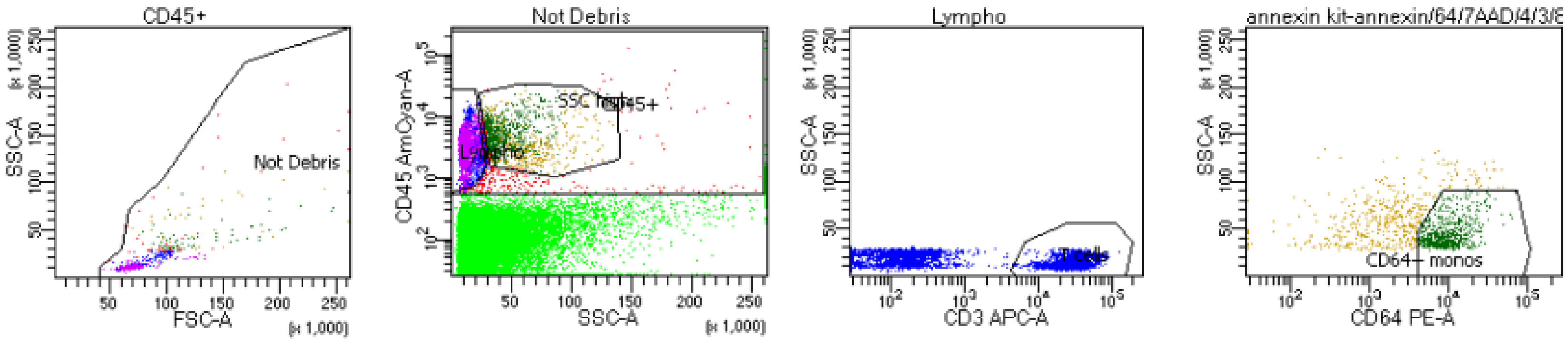

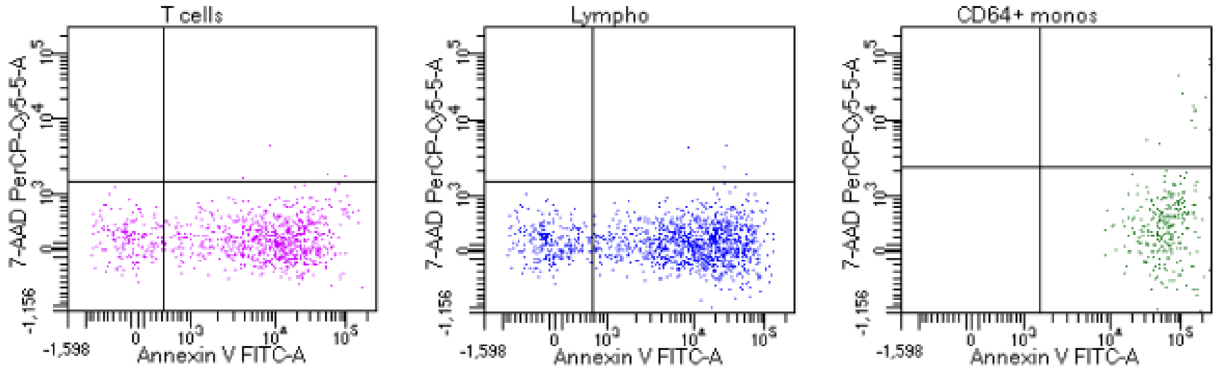

Population	%Parent
Necrotic - T cells	0.0
Late apoptotic - T cells	0.5
Live - T cells	18.0
Early apoptotic - T cells	81.5

Population	%Parent
Necrotic - lympho	0.0
Late apoptotic - lympho	0.4
Livec - lympho	18.7
Early apoptotic - lympho	80.8

Population	%P...
Necrotic - monocytes	0.0
Late apoptotic - monocytes	3.4
Live - monocytes	0.0
Early apoptotic - monocytes	96.5

Tube: annexin/64/7AAD/4/3/8/47/45

Population

- All Events
  - Not Debris
    - CD45+
      - Lympho
        - Necrotic - lympho
        - Late apoptotic - lympho
        - Livec - lympho
        - Early apoptotic - lympho
      - T cells
        - Necrotic - T cells
        - Late apoptotic - T cells
        - Live - T cells
        - Early apoptotic - T cells
      - SSC high
        - CD64+ monos
          - Necrotic - monocytes
          - Late apoptotic - monocytes
          - Live - monocytes
          - Early apoptotic - monocytes

Patient 7 ECP MNC 1

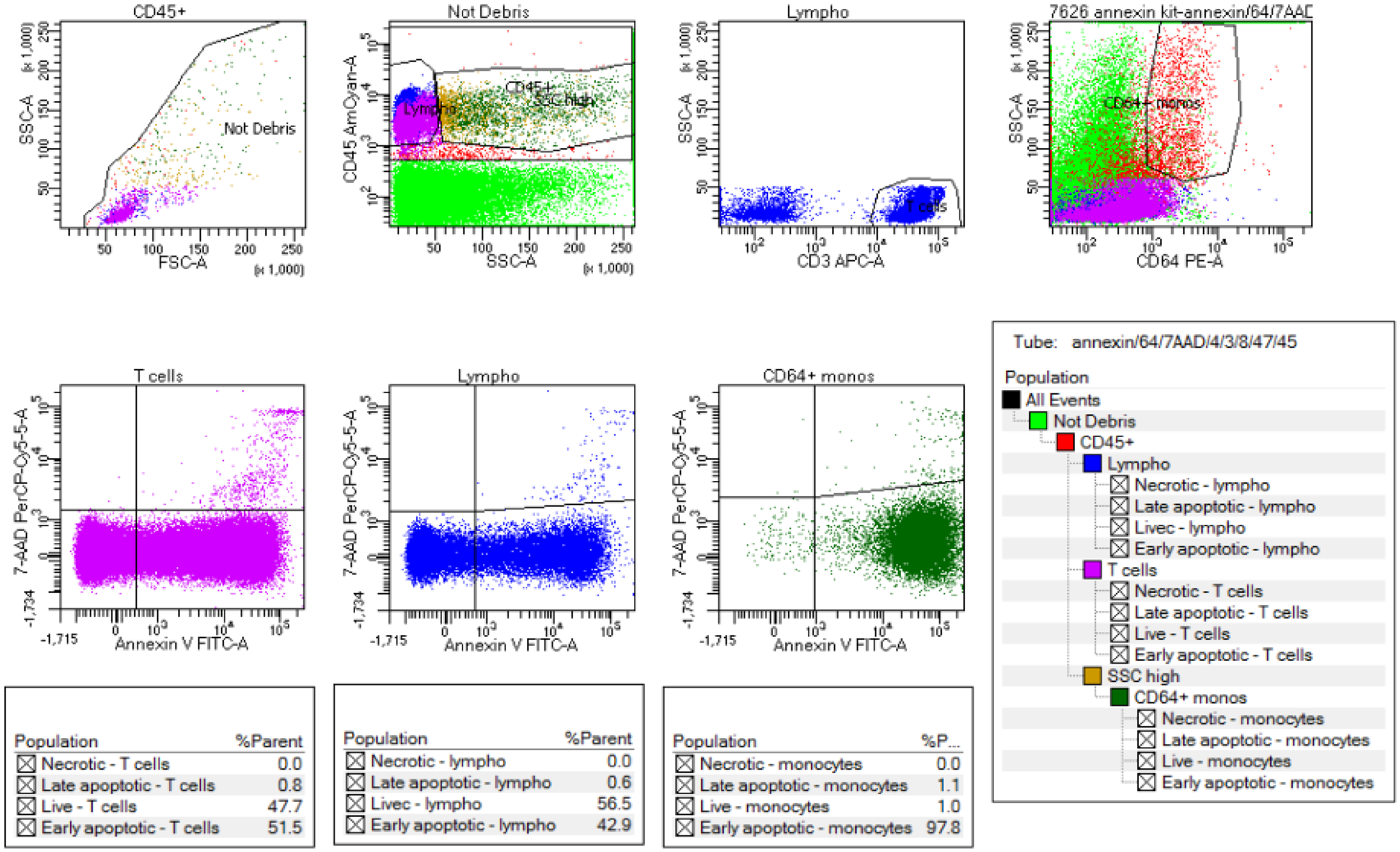

Population	%Parent
Necrotic - T cells	0.0
Late apoptotic - T cells	0.8
Live - T cells	47.7
Early apoptotic - T cells	51.5

Population	%Parent
Necrotic - lympho	0.0
Late apoptotic - lympho	0.6
Livec - lympho	56.5
Early apoptotic - lympho	42.9

Population	%P...
Necrotic - monocytes	0.0
Late apoptotic - monocytes	1.1
Live - monocytes	1.0
Early apoptotic - monocytes	97.8

Patient 7 ECP MNC 2

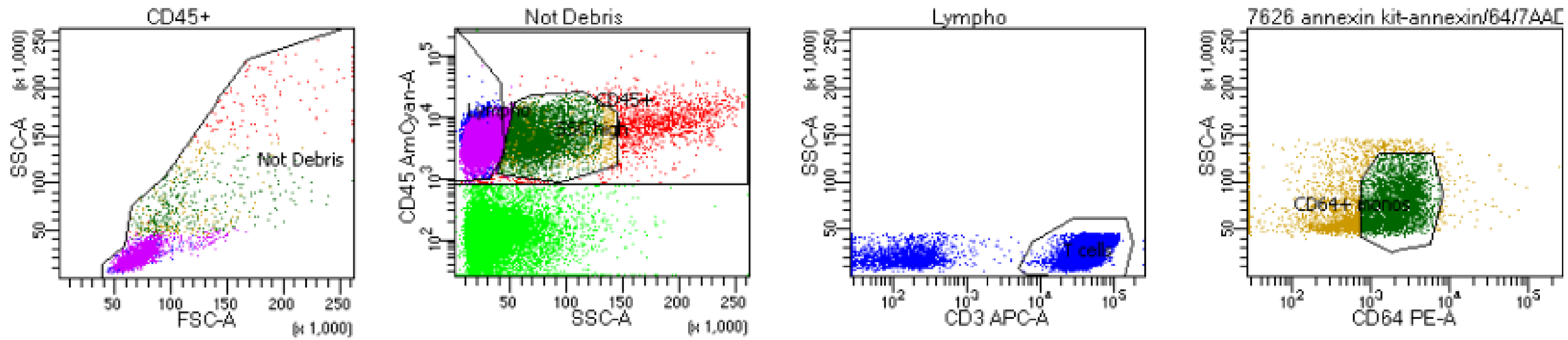

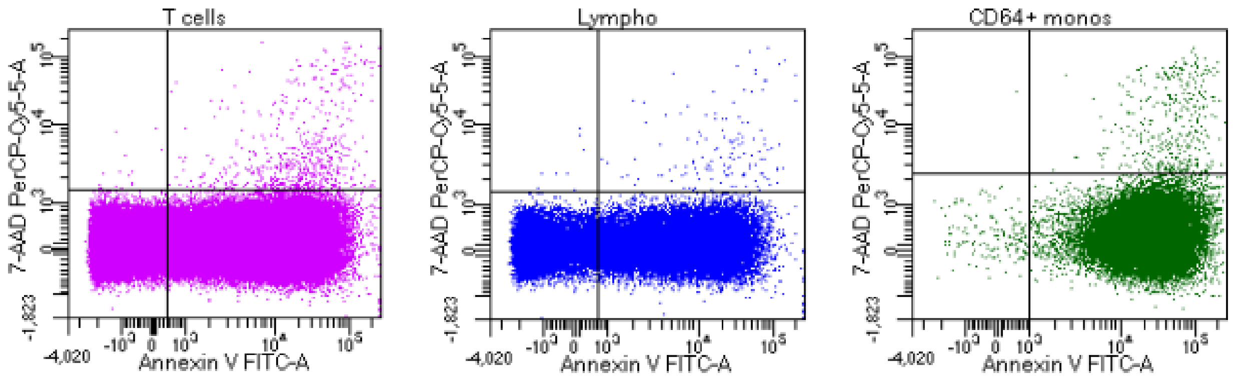

Population	%Parent
☒ Necrotic - T cells	0.0
☒ Late apoptotic - T cells	0.4
☒ Live - T cells	32.9
☒ Early apoptotic - T cells	66.8

Population	%Parent
☒ Necrotic - lympho	0.0
☒ Late apoptotic - lympho	0.3
☒ Livec - lympho	41.4
☒ Early apoptotic - lympho	58.3

Population	%P...
☒ Necrotic - monocytes	0.0
☒ Late apoptotic - monocytes	1.1
☒ Live - monocytes	0.5
☒ Early apoptotic - monocytes	98.4

Tube: annexin/64/7AAD/4/3/8/47/45

Population

- All Events
  - Not Debris
    - CD45+
      - Lympho
        - ☒ Necrotic - lympho
        - ☒ Late apoptotic - lympho
        - ☒ Livec - lympho
        - ☒ Early apoptotic - lympho
      - T cells
        - ☒ Necrotic - T cells
        - ☒ Late apoptotic - T cells
        - ☒ Live - T cells
        - ☒ Early apoptotic - T cells
      - SSC high
        - CD64+ monos
          - ☒ Necrotic - monocytes
          - ☒ Late apoptotic - monocytes
          - ☒ Live - monocytes
          - ☒ Early apoptotic - monocytes

Patient 7 ECP MNC 3

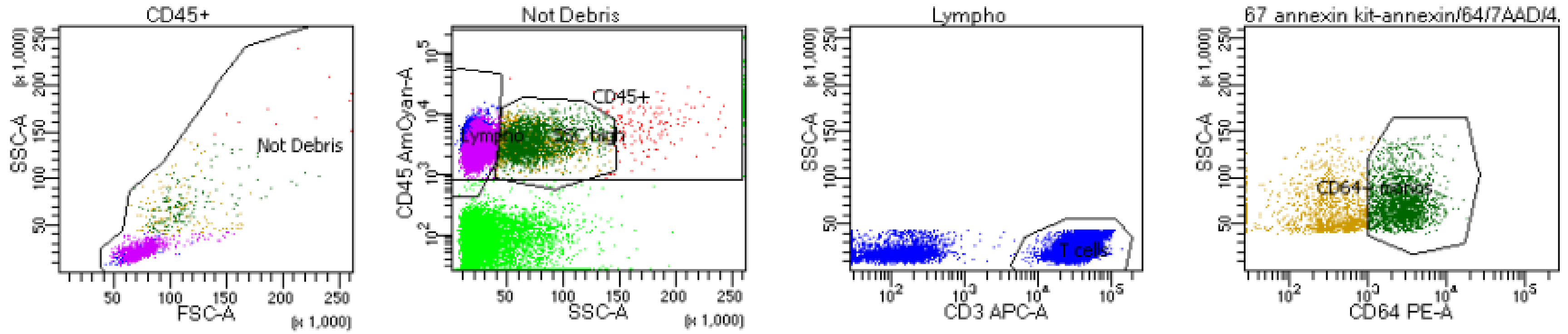

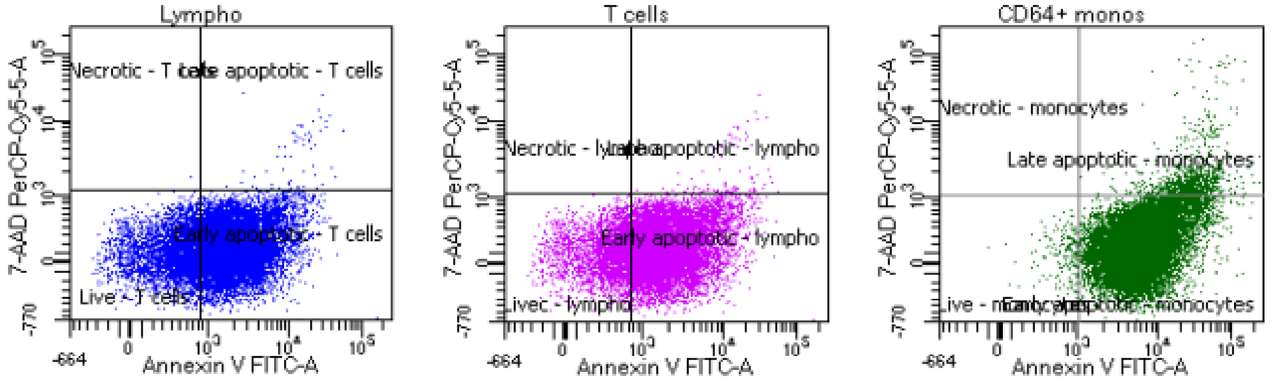

Population	%Parent
Necrotic - T cells	0.0
Late apoptotic - T cells	0.6
Live - T cells	26.7
Early apoptotic - T cells	72.7

Population	%Parent
Necrotic - lympho	0.0
Late apoptotic - lympho	0.7
Livec - lympho	23.7
Early apoptotic - lympho	75.6

Population	%P...
Necrotic - monocytes	0.0
Late apoptotic - monocytes	5.4
Live - monocytes	1.1
Early apoptotic - monocytes	93.5

Tube: annexin/64/7AAD/4/3/8/47/45

Population

- All Events
  - Not Debris
    - CD45+
      - Lympho
        - Necrotic - lympho
        - Late apoptotic - lympho
        - Livec - lympho
        - Early apoptotic - lympho
      - T cells
        - Necrotic - T cells
        - Late apoptotic - T cells
        - Live - T cells
        - Early apoptotic - T cells
      - SSC high
        - CD64+ monos
          - Necrotic - monocytes
          - Late apoptotic - monocytes
          - Live - monocytes
          - Early apoptotic - monocytes

Patient 7 ECP MNC 4

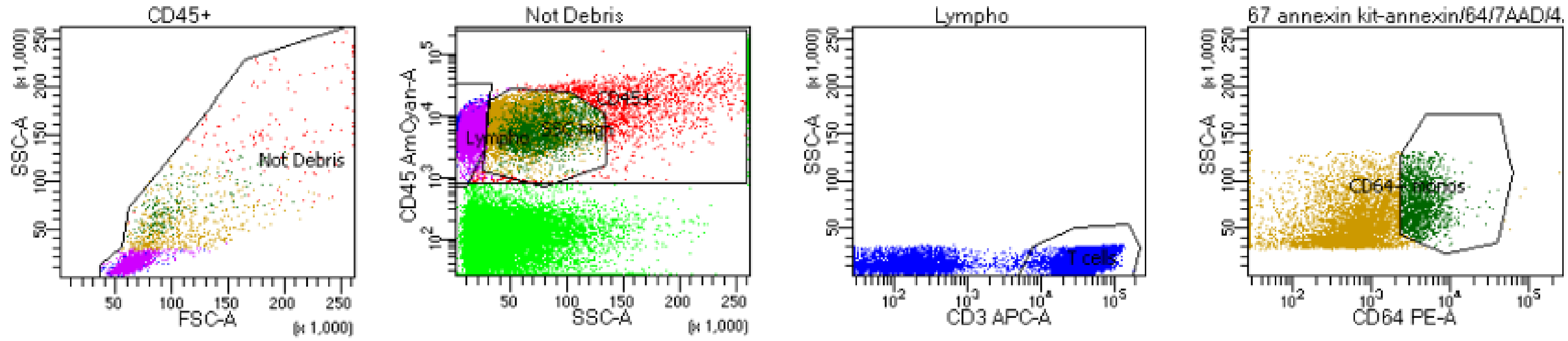

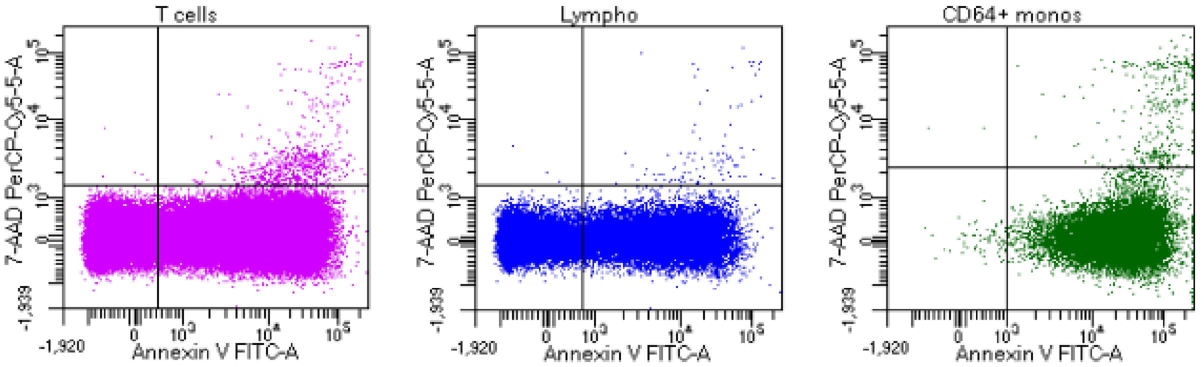

Population	%Parent
Necrotic - T cells	0.0
Late apoptotic - T cells	0.5
Live - T cells	32.4
Early apoptotic - T cells	67.0

Population	%Parent
Necrotic - lympho	0.0
Late apoptotic - lympho	0.3
Livec - lympho	47.1
Early apoptotic - lympho	52.6

Population	%P...
Necrotic - monocytes	0.0
Late apoptotic - monocytes	2.3
Live - monocytes	0.5
Early apoptotic - monocytes	97.2

Tube: annexin/64/7AAD/4/3/8/47/45

Population

- All Events
  - Not Debris
    - CD45+
      - Lympho
        - Necrotic - lympho
        - Late apoptotic - lympho
        - Livec - lympho
        - Early apoptotic - lympho
      - T cells
        - Necrotic - T cells
        - Late apoptotic - T cells
        - Live - T cells
        - Early apoptotic - T cells
      - SSC high
        - CD64+ monos
          - Necrotic - monocytes
          - Late apoptotic - monocytes
          - Live - monocytes
          - Early apoptotic - monocytes
