# Supplementary material for: Extracorporeal Photopheresis as a Possible Therapeutic Approach for Adults with Severe and Critical COVID-19 Non-Responsive to Standard Treatment: A Pilot Investigational Study
Source: J Clin Med. 2023 Jul 29;12(15):5000. doi: 10.3390/jcm12155000 (PMC10420323; doi:10.3390/jcm12155000)
Supplement: Supplementary file 1 [file jcm-12-05000-s001.zip › Table S1.pdf]

SUPPLEMENTARY TABLE

Supplementary Table S1. Schematic diagram for data acquisition at baseline, inclusion and follow-up.

**PARAMETERS**	**Baseline**	**Inclusion**	**ECP sessions**				**Post-ECP follow-up**			
			**1st**	**2nd**	**3rd**	**4th**	**+3 days**	**+7 days**	**+14 days**	**+28 days**
**1. Clinical assessments**										
Physical examination	x	x	x	x	x	x	x	x	x	x
Arterial blood gas analysis	x	x	x	x	x	x	x	x	x	x
Requirement of ICU admission	x	x	x	x	x	x	x	x	x	x
Requirement of hospitalization	x	x	x	x	x	x	x	x	x	x
Requirement of endotracheal intubation & invasive mechanical ventilation	x	x	x	x	x	x	x	x	x	x
Survival assessment	x	x	x	x	x	x	x	x	x	x
Concomitant medications	x	x	x	x	x	x	x	x	x	x
Adverse events	x	x	x	x	x	x	x	x	x	x
Appearance of secondary infections, ARDS or cytokine storm	x	x	x	x	x	x	x	x	x	x
**2. Radiological assessment**										
Chest CT scan with intravenous contrast	x	x					x	x	x	x
**3. Complex laboratory assessments**										
A) Routine laboratory studies										
Complete blood counts	x	x	x	x	x	x	x	x	x	x
Serum chemistry	x	x	x	x	x	x	x	x	x	x
Blood inflammatory markers (plasma IL-6, serum CRP, PCT, ferritin)	x	x	x	x	x	x	x	x	x	x
Coagulation tests (INR, aPTI, TT, plasma fibrinogen and D-dimer)	x	x	x	x	x	x	x	x	x	x
B) Virological markers*										
Respiratory SARS-CoV-2 RT-PCR	x	x					x	x	x	x
Peripheral whole blood SARS-CoV-2 RT-PCR	x	x					x	x	x	x
C) Immune biomarker profiling										
Multi-cytokine analysis: serum IFN-α, IFN-γ, IP-10, MCP-1, MIP-1α, RANTES, TNF-α, TNF-β, plasma IL-1α, IL-1β, IL-2, IL-4, IL-5, IL-6, IL-8, IL-10, IL-12, IL-13, IL-15, IL-17A	x	x					x	x	x	x
Serum electrophoresis with immunofixation	x	x					x	x	x	x
Serum IgA, IgM and IgG	x	x					x	x	x	x
Serum C3 and C4	x	x					x	x	x	x
Serum autoantibodies ANA, ENA, anti-dsDNA, ANCA	x	x					x	x	x	x
Peripheral whole blood fluorescence-activated cell sorting (FACS)**	x	x					x	x	x	x
D) Assessment of the ECP MNC*										
ECP MNC fluorescence-activated cell sorting (FACS)**			x	x	x	x				
ECP MNC Multi-cytokine analysis			x	x	x	x				

ANA: anti-nuclear antibody, ANCA: anti-neutrophil cytoplasmic antibody, aPTT: activated partial thromboplastin time, ARDS: acute respiratory distress syndrome, C: complement, CT: computed tomography, CRP: C-reactive protein, dsDNA: double-stranded deoxyribonucleic acid, ECP: extracorporeal photopheresis, ENA: extractable nuclear antigen, FACS: fluorescence-activated cell sorting, ICU: intensive care unit, IFN: interferon, Ig: immunoglobuline, IL: interleukin, INR: international normalized ratio, PCT: procalcitonin, RT-PCR: real-time polymerase chain reaction, SARS-CoV-2: severe acute respiratory syndrome coronavirus 2, TT: thrombin time.

* PCR was performed by using the Allplex^{TM} SARS-CoV-2 RT-PCR Assay (Seegene Inc.).

** Lymphocyte subpopulation percentages and absolute cell numbers were given using the BD Multitest 6-color TBNK kit (reference number 337166, BD Biosciences). For identification of regulatory T cells, we used the HU Regulatory T-cell cocktail (ref. no. 560249, BD Biosciences). For identification of apoptotic and necrotic cells, the Annexin V-FITC Kit was used (ref. no. 130-092-052, Miltenyi Biotec). Other T cell parameters were given with the use of individual markers, using the lyse–wash methodology. The following reagents were used: CD25 FITC (ref. no. 345796, BD Biosciences), CD45 RA PC5.5 (ref. no. 563429, BD Biosciences), CD45 RO PE (ref. no. 555493, BD Biosciences) CD3 APC (ref. no. 345767, BD Biosciences) CD4 PacificBlue (ref. no. 558116, BD Biosciences), TCR-alpha/beta FITC (ref. no. 333140 BD Biosciences), TCR-gamma/delta-1 PE (ref. no. 333141, BD Biosciences), HLA-DR PerCP (ref. no. 347402, BD Biosciences), CD8 Vio-770 (ref. 130-113-155, Miltenyi Biotec), CD45 Chrome Orange (ref. no. B36294, Beckman Coulter). Cytokines were analyzed simultaneously with bead-based fluorometric xMAP® technology, using MILLIPLEX® Human Cytokine/Chemokine/Growth Factor Panel on Luminex® Magpix (Sigma-Aldrich).
