# Supplementary material for: Extracorporeal Photopheresis as a Possible Therapeutic Approach for Adults with Severe and Critical COVID-19 Non-Responsive to Standard Treatment: A Pilot Investigational Study
Source: J Clin Med. 2023 Jul 29;12(15):5000. doi: 10.3390/jcm12155000 (PMC10420323; doi:10.3390/jcm12155000)
Supplement: Supplementary file 1 [file jcm-12-05000-s001.zip › Table S2.pdf]

SUPPLEMENTARY TABLE

Supplementary Table S2. FACS with early and late apoptosis markers and multi-cytokine analysis from ECP mononuclear cell collections (MNC) after ECP sessions 1 (first) to 4 (last) of patients enrolled in the study.

PARAMETER	ECP MNC1	ECP MNC2	ECP MNC3	ECP MNC4	*p* value*
**Regulatory T cell ratio (%)**	0.5±0.5 (0.2–1.0)	0.5±0.7 (0.2–2.3)	0.3±0.3 (0.1–0.7)	0.4±1.2 (0.1–2.1)	0.93
**CD3+ T cell count (cells/µl)**	3059±3843 (708–18449)	3275±2679 (860–24117)	1955±6271 (726–35996)	2184±5632 (302–21951)	0.72
**CD3+/CD8+ T cell count (cells/µl)**	1932±1606 (246–4177)	1942±1235 (301–3191)	1093±1179 (343–2932)	1098±1457 (135–2371)	0.87
**CD3+/CD4+ T cell count (cells/µl)**	2064±2008 (347–16481)	1420±1694 (260–22082)	875±4995 (287–32992)	873±4500 (159–19468)	0.49
**CD3-/CD16+/CD56+ NK cell count (cells/µl)**	232±242 (105–2231)	186±245 (59–2160)	160±88 (66–761)	85±78 (42–708)	0.26
**CD19+ B cell count (cells/µl)**	237±1126 (0–2349)	211±1304 (0–2464)	562±2691 (0–5074)	764±2601 (0–4346)	0.79
**Double negative T cell ratio (%)**	2.3±2.8 (0.1–13.1)	1.3±1.6 (0.2–8.4)	1.1±1.3 (0.1–6.1)	0.7±0.8 (0.2–4.5)	0.81
**Double positive T cell ratio (%)**	0.1±0.2 (0–1.3)	0.1±0.1 (0–1.3)	0.1±0.1 (0–1.1)	0.1±0.3 (0–1.3)	0.58
**Counts within the CD3+ gate:**					
CD3+/TCRab+ T cell (cells/µl)	2734±1360 (707–5215)	3521±1400 (8464620)	1030±9029 (689–35888)	1167±6773 (292–21864)	0.84
CD3+/TCRgd+ T cell (cells/µl)	65±152 (0.9–228)	123±52 (43–149)	40±57 (2–82)	8±29 (0.3–78)	0.3
CD3+/CD25+ T cell (cells/µl)	375±323 (42–763)	119±778 (25–1582)	189±256 (17–467)	89±199 (9–258)	**0.04**
CD3+/HLA-DR+ T cell (cells/µl)	292±341 (52–1005)	267±815 (65–1696)	424±1414 (43–5378)	253±283 (0.1–357)	0.67
**Counts within the CD4+ gate:**					
CD4+/CD45RA+ naive T cell (cells/µl)	200±1029 (29–1408)	717±316 (88–813)	96±7708 (33–30815)	104±2557 (39–17579)	0.12
CD4+/CD45RO+ memory T cell (cells/µl)	465±554 (190–1494)	1000±631 (173–1599)	449±787 (202–2177)	604±1487 (119–3710)	0.88
**Counts within the CD8+ gate:**					
CD8+/CD45RA+ naive T cell (cells/µl)	848±1365 (152–1973)	1042±1362 (469–2581)	354±287 (146–703)	415±347 (95–770)	0.52
CD8+/CD45RO+ memory T cell (cells/µl)	243±293 (81–654)	330±123 (146–584)	240±718 (85–2234)	251±504 (39–1864)	0.88
**Lymphocyte apoptosis markers (%):**					
- Live lymphocytes	64.4±15.5 (0.6–81.6)	59.2±35.5 (41.4–96.9)	54.3±24.7 (23.7–79.8)	52.3±14.5 (41.4–86.6)	0.81
- Necrotic lymphocytes	0	0±0.1 (0–0.5)	0	0	n.a.
- Early apoptotic lymphocytes	35.1±15.9 (18.4–98.1)	39.9±35.7 (3.0–58.3)	43.5±23.6 (19.6–75.6)	51.2±11.5 (34.6–129.0)	0.74
- Late apoptotic lymphocytes	0.6±0.5 (0–1.3)	0.4±0.4 (0.1–0.9)	0.4±0.5 (0.1–4.3)	0.4±0.3 (0.1–1.0)	0.93
**T cell apoptosis markers (%):**					
- Live T cells	60.8±25.8 (2.1–72.1)	59.6±35.0 (32.9–94.5)	61.4±30.2 (26.7–83.4)	51.7±32.2 (32.4–87.9)	0.41
- Necrotic T cells	0	0±0.1 (0–1.0)	0	0	n.a.
- Early apoptotic T cells	38.7±26.0 (27.8–97.7)	39.4±35.3 (5.3–66.8)	35.8±28.8 (16.0–72.7)	47.6±32.8 (11.8–67.0)	0.55
- Late apoptotic T cells	0.5±0.5 (0–0.9)	0.4±0.4 (0.2–1.0)	0.4±0.4 (0.1–5.2)	0.5±0.5 (0.1–1.0)	0.93
**Monocyte apoptosis markers (%):**					
- Live monocytes	0.4±2.6 (0–14.8)	0.5±0.5 (0–74)	0.7±0.8 (0.1–10.6)	0.5±12.5 (0.2–30.1)	0.41
- Necrotic monocytes	0	0	0	0	n.a.
- Early apoptotic monocytes	97.8±7.6 (84.0–99.5)	97.6±6.2 (25.2–98.5)	95.2±5.3 (88.8–99.4)	95.9±13.9 (68.2–99.0)	0.68
- Late apoptotic monocytes	1.3±1.2 (0.4–10.4)	1.4±1.2 (0.6–12.7)	1.5±3.9 (0.5–6.5)	1.8±1.2 (0.6–4.9)	0.5
**Serum interferon-α (pg/ml)**	24.3±19.8 (8.0–36.5)	32.6±31.0 (8.–55.5)	11.4±10.5 (8.0–55.5)	8.0±12.2 (3.4–39.3)	0.1
**Serum interferon-γ (pg/ml)**	1.3±0.8 (1.3–4.5)	1.3±2.0 (1.3–5.6)	1.3±0.1 (1.3–5.0)	1.3±1.9 (1.3–11.8)	0.75
**Plasma interleukin-1α (pg/ml)**	4.8±8.1 (4.8–45.4)	4.8±9.7 (4.8–36.8)	8.8±69.2 (4.8–221.8)	11.0±7.7 (4.8–32.9)	0.09
**Plasma interleukin-1β (pg/ml)**	1.6±0.5 (1.6–7.7)	1.6±1.4 (1.6–4.7)	1.6±0.1 (1.6–4.7)	1.6±0.1 (1.6–6.4)	0.56
**Plasma interleukin-2 (pg/ml)**	0.6±0.1 (0.6–1.0)	0.6±0.1 (0.6–0.7)	0.6±0.1 (0.6–0.8)	0.6±0.1 (0.6–1.4)	1.0
**Plasma interleukin-4 (pg/ml)**	0.7±0.5 (0.6–2.8)	1.0±3.2 (0.6–8.6)	0.6±0.3 (0.6–1.6)	0.6±0.3 (0.6–1.2)	0.39
**Plasma interleukin-5 (pg/ml)**	4.0±8.7 (0.7–32.7)	4.5±3.2 (0.6–8.3)	1.7±1.2 (0.6–5.1)	3.5±3.9 (1.0–5.9)	0.68

**Plasma interleukin-6 (pg/ml)**	102.1±191.1 (7.7–7005.0)	58.2±107.5 (4.0–863.2)	51.5±386.8 (7.9–2292.7)	30.2±486.7 (9.7–4411.9)	0.81
**Plasma interleukin-8 (pg/ml)**	4.8±7.8 (0.9–20.2)	3.3±6.5 (0.6–14.6)	4.2±3.7 (0.6–9.6)	3.0±2.2 (0.8–6.3)	0.36
**Plasma interleukin-10 (pg/ml)**	12.6±8.2 (2.6–28.7)	10.5±7.4 (2.6–17.3)	16.4±40.6 (2.6–92.1)	13.0±40.8 (2.6–91.7)	0.93
**Plasma interleukin-12 (pg/ml)**	3.0±0.1 (3.0–3.0)	3.0±1.3 (3.0–6.2)	3.0±0.7 (3.0–7.0)	3.0±0.7 (3.0–18.2)	0.24
**Plasma interleukin-13 (pg/ml)**	16.1±14.5 (6.4–48.3)	10.7±35.4 (6.4–63.5)	6.4±7.3 (6.4–63.5)	6.4±7.3 (6.4–31.4)	0.32
**Plasma interleukin-15 (pg/ml)**	4.5±4.1 (3.0–11.5)	4.1±3.0 (3.0–10.3)	4.1±3.9 (3.0–8.9)	5.7±4.8 (3.0–8.4)	0.95
**Plasma interleukin-17A (pg/ml)**	1.3±2.2 (1.3–5.2)	2.4±3.3 (1.3–6.1)	1.3±0.1 (1.3–2.7)	1.3±0.1 (1.3–2.7)	0.42
**Serum IP-10 (pg/ml)**	488.5±1777.0 (48.7–10731.5)	263.7±767.5 (51.3–1269.4)	153.4±205.7 (33.6–487.6)	121.8±549.1 (52.1–1567.4)	0.23
**Serum MCP-1 (pg/ml)**	188.5±258.0 (78.9–2401.7)	164.6±90.4 (90.0–479.4)	147.8±139.8 (23.5–497.3)	179.2±204.9 (25.9–471.1)	0.75
**Serum MIP-1α (pg/ml)**	15.1±15.2 (3.0–25.1)	13.1±2.9 (3.0–26.2)	10.6±15.4 (3.0–22.9)	8.3±20.8 (3.0–34.5)	0.56
**Serum RANTES (pg/ml)**	1093.8±1235.3 (650.0–2623.2)	1100.8±274.3 (730.8–2149.5)	1416.5±1025.7 (383.2–2608.6)	1107.1±416.1 (93.3–2250.2)	0.97
**Serum TNF-α (pg/ml)**	19.2±32.0 (13.0–134.6)	22.7±25.6 (8.7–46.3)	20.0±60.5 (9.1–153.6)	21.8±68.1 (11.5–193.1)	0.52
**Serum TNF-β (pg/ml)**	4.8±8.2 (1.6–15.5)	1.6±18.1 (1.6–21.9)	1.6±0.1 (1.6–9.8)	1.6±1.3 (1.6–6.6)	0.18

* *p* values are calculated between data measured from ECP MNC1 (first session) and ECP MNC4 (last session).

n.a.: not applicable
