# Supplementary figures and images for: Extracorporeal Photopheresis as a Possible Therapeutic Approach for Adults with Severe and Critical COVID-19 Non-Responsive to Standard Treatment: A Pilot Investigational Study
Source: J Clin Med. 2023 Jul 29;12(15):5000. doi: 10.3390/jcm12155000 (PMC10420323; doi:10.3390/jcm12155000)

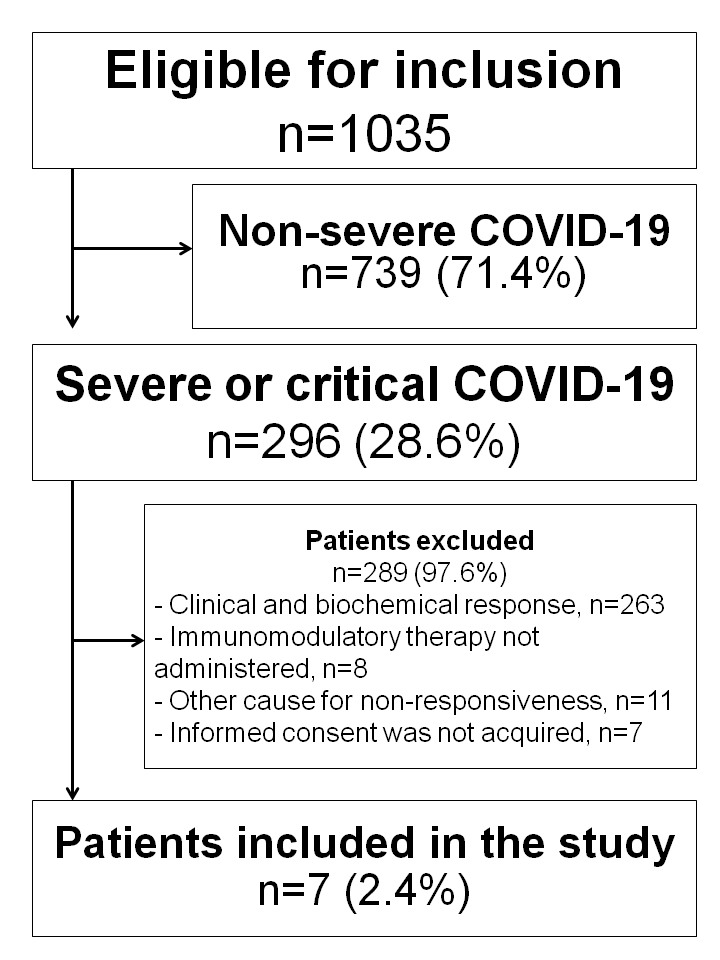

Supplement: Supplementary file 1 [file jcm-12-05000-s001.zip › Figure S1.tif]
